# A study on a privacy preservation method for VANETs based on certificateless anonymous authentication and non-interactive zero-knowledge proof

**DOI:** 10.1371/journal.pone.0358272

**Published:** 2026-10-27

**Authors:** Zhaowei Hu, Hangyi Quan, ShiYun Ni, Peng He

**Affiliations:** School of Computing Science and Artificial Intelligence, Changzhou University, Changzhou, China; Jaramogi Oginga Odinga University of Science and Technology, KENYA

## Abstract

As a core component of intelligent transportation systems, the security authentication and privacy protection mechanisms in Vehicular Ad Hoc Networks (VANETs) are of paramount importance. To address key security issues in VANETs, such as the vulnerability of identity privacy to leakage, the complexity of certificate management, the high risk of key escrow, and the challenge of balancing authentication efficiency with security, the paper proposes a privacy-preserving identity authentication and key agreement method based on certificateless public key cryptography (CL-PKC) and non-interactive zero-knowledge proofs (NIZK). This method utilizes a composite identity identifier and combines it with Pedersen commitments to propose a dynamic anonymous identity generation mechanism. It stores static commitments containing identity information on the blockchain, and generates one-time dynamic identities through re-randomization in each authentication process, thereby solving the certificate management issue and achieving anonymity. Furthermore, the method designs an authentication protocol based on CL-PKC and Schnorr NIZK, integrating private key validity verification with dynamic identity verification. A vehicle only needs to construct a single zero-knowledge proof to complete both verifications simultaneously, eliminating the risk of key escrow and improving authentication efficiency and security. For experimental evaluation, the paper constructs a simulation environment based on standard elliptic curve cryptography (ECC) and the Ganache blockchain testnet, utilizing benchmark testing data to conduct a comprehensive performance comparison between the proposed method and existing typical authentication methods. Security analysis and experimental evaluation demonstrate that the proposed method satisfies security requirements such as conditional anonymity, unlinkability, and forward security, effectively reduces the system’s computational and communication overhead, and is applicable to highly dynamic and resource-constrained VANET environments.

## 1. Introduction

With the rapid advancement of the automotive industry and wireless communication technologies, the development of intelligent transportation systems has become an inevitable trend [1]. VANETs interconnect vehicles with external networks, other vehicles, and infrastructure via wireless communication, facilitating real-time data exchange. They have emerged as a key enabler for intelligent transportation system applications [2]. In VANETs, vehicles collect various types of information through their own sensors, including traffic status, road conditions, vehicle locations, accident alerts, and emergency notifications. This information is transmitted via broadcast or peer-to-peer methods, where it is received and processed by other vehicles or infrastructure. Based on the received information, corresponding decisions are made, such as avoiding congested routes or adjusting driving speed. Through wireless communication and information exchange between vehicles, VANETs enable real-time collaboration and data sharing, which has significant application value in traffic safety, traffic flow optimization, and emergency services. It also helps in building a more intelligent, efficient, and secure transportation system [3].

However, the open nature of VANETs exposes transmitted messages to significant security threats. Firstly, communication messages are susceptible to various attacks, such as spoofing, replay, tampering, and signature forgery [4–6]. For example, a malicious vehicle can join the network to generate and broadcast false emergency messages, which could mislead others’ driving decisions and potentially cause safety hazards. Secondly, messages transmitted over open channels often contain sensitive personal information, such as license plate numbers and driver identities. If intercepted by malicious actors, this data could lead to financial losses for users and even pose threats to their personal safety [7]. Thirdly, traditional solutions based on Public Key Infrastructure (PKI) are widely used, but their reliance on digital certificates introduces significant certificate management overhead, communication bottlenecks, and risks of single points of failure.

In response to the above-mentioned issues, an increasing number of studies in recent years have begun exploring the application of blockchain technology to authentication frameworks. With its decentralized, tamper-proof, and traceable characteristics, blockchain can replace traditional centralized authentication authorities to securely store identity commitments, public key parameters, and other authentication information, providing an ideal foundation for building a secure identity authentication framework. Compared to lightweight distributed databases or log systems, blockchain, through multi-party joint maintenance and a distributed consensus mechanism, avoids excessive reliance on a single management node. By leveraging distributed ledger technology, it enables authentication evidence to be collectively maintained by all participants and stored across network nodes, ensuring the consistency and tamper-resistance of all authentication metadata. Even if some nodes are attacked or encounter anomalies, the distributed traceability of authentication data remains unaffected, thereby providing a higher level of security for VANETs compared to traditional databases. However, a pure blockchain solution still faces the challenge of implementing anonymous authentication and privacy protection without disclosing real identities. Therefore, achieving efficient, secure, and traceable anonymous identity authentication without compromising user privacy has become a critical issue to be addressed in VANETs.

The paper proposes a privacy-preserving identity authentication and key agreement method for VANETs based on CL-PKC and NIZK to address the identity authentication and privacy leakage issues faced in VANETs. The method eliminates the complexity of certificate management and the risk of key escrow inherent in traditional PKI systems by utilizing certificateless cryptography. By employing NIZK technology based on the Schnorr protocol, it ensures strong anonymity and zero-knowledge properties when vehicles prove their legitimate identity. Furthermore, the introduction of blockchain guarantees the immutability and auditability of all authentication metadata. The main contributions are as follows:

Firstly, a dynamic anonymous identity generation and authentication method is proposed based on Pedersen commitments and a self-designed composite identity identifier. The method effectively hides the vehicle’s real identity using the composite identity identifier (CID) and stores static commitments containing identity information on the blockchain. In each authentication process, a one-time dynamic identity is generated through re-randomization, addressing the certificate management issues and large-scale pseudonym maintenance overhead found in traditional PKI. It ensures anonymity and privacy protection for vehicles while enabling legitimate identity verification.

Secondly, an anonymous authentication and key agreement method is proposed based on CL-PKC and Schnorr NIZK. By integrating private key validity verification with dynamic identity verification, the method allows vehicles to construct a single zero-knowledge proof to simultaneously complete dual verification and securely negotiate a shared session key. This eliminates the risk of system key escrow and ensures the confidentiality and forward security of subsequent communications.

Thirdly, a comprehensive security analysis and performance evaluation are conducted based on formal security theory and a standard ECC simulation environment. The results show that the proposed method satisfies security requirements in VANETs, including identity anonymity, unlinkability, and resistance to physical attacks. The method also features low computational and communication overhead, making it suitable for highly dynamic and resource-constrained VANETs.

The remainder of the paper is organized as follows: Section 2 reviews related work on security and privacy in VANETs. Section 3 details the system model, trust assumptions, threat model, and security objectives. Section 4 describes the proposed authentication method in detail. Section 5 provides a systematic security analysis of the proposed method. Section 6 evaluates the performance of the proposed method through experiments and comparative analysis. Section 7 concludes the paper and outlines future work.

## 2. Related work

In VANETs, vehicles communicate with each other and with RSUs via wireless channels. In an open network environment without adequate security protection, the risk of information leakage or tampering increases significantly. Therefore, constructing an effective identity authentication mechanism is a critical prerequisite for ensuring secure communication in VANETs. Current authentication schemes in VANETs primarily revolve around zero-knowledge proofs, CL-PKC, and blockchain technology, achieving identity management, privacy protection, and trust construction at various levels.

### 2.1 Zero-knowledge proof-based methods

In VANETs, traditional authentication schemes based on PKI or Identity-Based Cryptography (IBC) typically require transmitting digital certificates or pseudonyms over open channels. This still poses a risk of privacy leakage when facing advanced eavesdropping and trajectory tracking attacks. Zero-knowledge proof (ZKP) technology allows a prover to convince a verifier of its legitimacy without disclosing any useful information to the verifier.

Due to the strong privacy-preserving characteristics of zero-knowledge proof methods, ZKP technology has attracted extensive attention and in-depth research from scholars worldwide in the field of anonymous identity authentication in VANETs in recent years. In terms of lightweight designs to reduce communication and computation overheads, Xi et al. [8] proposed an anonymous mutual authentication scheme (ZAMA) for VANETs based on ZKP and ECC. This scheme introduces a lightweight ZKP verification mechanism, effectively reducing the computational cost of mutual authentication between vehicles and cloud servers. However, its complex operations during the initial authentication still incur certain computational delays. Furthermore, it fails to achieve seamless cross-domain handovers when a vehicle enters the coverage area of a new roadside unit (RSU). Kalmykov et al. [9] proposed an adaptive zero-knowledge identity authentication protocol suitable for VANETs. This scheme dynamically adjusts the privacy protection level of authentication based on changes in road traffic density, thereby effectively reducing authentication latency. Nevertheless, it overly relies on dynamic parameter adjustments. In extreme scenarios with drastic traffic fluctuations, its security lower bound is difficult to guarantee with strict cryptographic proofs. To further reduce the number of communication rounds, Li et al. [10] proposed a sustainable and round-optimized group authentication key exchange scheme for vehicular communication. This method effectively decreases the interactive communication rounds between vehicles and infrastructure (V2I), improving the efficiency of two-factor authentication. However, when handling vehicles frequently crossing the coverage areas of different RSUs, the computational and communication overheads of repeated authentication caused by its intra-group key management and authentication mechanisms remain high. To integrate ZKP with decentralized schemes and address the vulnerability bottleneck of traditional centralized Trusted Authorities (TAs), Jiang et al. [11] proposed a ZKP-based anonymous authentication scheme utilizing a main-side chain architecture (TRUG-PBFT) and lattice cryptography. This method not only employs lattice-based ZKP technology to resist potential future quantum computing threats but also uses the main-side chain to distribute the storage pressure of authentication data, significantly improving throughput in high-concurrency scenarios. Furthermore, Jiang et al. [12] proposed a distributed privacy-preserving method for VANETs combining ZKP and proxy re-encryption. This scheme ingeniously integrates ZKP with proxy re-encryption, blockchain, and the Inter Planetary File System (IPFS). It not only achieves vehicle identity anonymity but also ensures the secure sharing of driving data among distributed nodes. However, its proxy re-encryption mechanism is limited to static data access control and sharing.

### 2.2 Certificateless public key cryptography-based methods

An identity authentication scheme based on certificateless signatures was first proposed in 2003 [13]. Unlike PKI-based schemes, this type of scheme no longer relies on certificates to ensure the validity of public keys. By eliminating the certificate mechanism, it can effectively reduce communication, computation, and storage overhead [14]. Certificateless public key cryptography (CL-PKC), by dividing the private key into a system-generated component and a user-held component, can effectively address the key escrow problem and has been widely used in the design of VANET authentication schemes in recent years. Horng et al. [15] proposed a certificateless aggregate signature method for vehicle communications based on certificateless signatures. The method combines the advantages of certificateless cryptography and aggregate signatures, achieving anonymous authentication, message integrity, and unlinkability while balancing privacy protection with identity traceability. Cui et al. [16] introduced an elliptic curve cryptography-based certificateless aggregate signature method that enables secure communication in VANETs with conditional privacy protection. Its core mechanism conceals identities by mapping broadcast messages to pseudonyms, while enabling authorized tracing of real identities during disputes. The method outperforms bilinear pairing-based methods in computational efficiency but incurs storage and update overhead for pseudonym management. Li et al. [17] designed an efficient ECC-based certificateless short signature method with conditional privacy preservation. The method resists spoofing and tampering attacks while maintaining strong unlinkability. By avoiding computationally expensive bilinear pairing operations, it achieves lower computational and communication overhead than comparable methods, making it more suitable for resource-constrained vehicular environments. Similarly, in pursuit of communication efficiency, Qian et al. [18] proposed an efficient certificateless aggregate signature scheme based on conditional privacy protection. This scheme significantly reduces bandwidth and computational overhead using full aggregation techniques, and its security against adversarial attacks is proven under the Diffie-Hellman assumption. As the security requirements of VANETs deepen, researchers have started to focus on more complex threats, such as key leakage and internal attacks. Qiao et al. [19] developed a forward-secure authentication protocol supporting temporary identities and certificateless aggregate signatures, which provides good traceability and resistance to key exposure. Liu et al. [20] proposed a certificateless anonymous authentication and key agreement protocol, which innovatively separates identity authentication from traffic message verification, effectively avoiding the overhead of frequently checking message revocation lists. To address internal attack threats, Wang et al. [21] designed a certificateless, anonymous, and revocable authentication protocol. This construction not only achieves “single authentication, efficient broadcasting” but also effectively resists collusion and forgery attacks from malicious Private Key Generators (PKGs) and RSUs, making it highly suitable for privacy-sensitive VANET applications.

### 2.3 Blockchain-based methods

Blockchain-based privacy-preserving authentication methods for VANETs primarily build upon the PKI framework. Certificate Authorities issue digital certificates to vehicles, and the certificate information is distributed and stored across blockchain nodes. Blockchain technology provides decentralized and transparent services, ensuring that once information is stored, it becomes immutable and visible to all participants. This enables trustworthy and decentralized certificate management for VANETs. Yang et al. [22] proposed a blockchain-based traffic event verification method that replaces Proof-of-Work with Proof-of-Event consensus. RSUs collect regional traffic information, neighboring vehicles verify its authenticity, and confirmed data is uploaded to the blockchain. The method also enables the sending of warning messages to vehicles. However, each entity must validate event correctness, which increases verification latency and system time costs. Ali et al. [23] proposed a certificateless public-key signature scheme, which uses bilinear pairings to realize signature authentication and leverages blockchain technology to reveal and revoke the identities of malicious vehicles. This scheme offers high security and can resist replay attacks, tampering attacks, and other similar attacks, but the bilinear pairing operations lead to relatively high computational overhead. Zhou et al. [24] designed a novel conditional privacy-preserving authentication method integrating zero-knowledge signatures with blockchain technology. Vehicles use zero-knowledge signatures for signing and authentication, with all records stored on the blockchain to ensure traceability. However, the method achieves unlinkability only during the registration phase, failing to adequately protect user privacy.

In addition to the aforementioned schemes based on traditional cryptography or blockchain, researchers have proposed several inspiring lightweight schemes for the highly dynamic and resource-constrained Internet of Drones (IoD) environments in recent years. For instance, Tanveer et al. [25] proposed an authentication and key management protocol (RAMP-IoD) based on ECC and hash functions, which effectively reduces computation and communication overheads while ensuring the security of session keys. To further pursue efficiency, Tanveer et al. subsequently abandoned public-key operations and designed an anonymous authentication framework (SAAF-IoD) using chaotic maps and symmetric encryption [26]. This framework resists drone capture and ground station bypass attacks at extremely low computational costs. Furthermore, aiming at physical security threats in extreme environments, Tanveer et al. [27] introduced Physical Unclonable Functions (PUFs) and the AEGIS authenticated encryption scheme to propose the PAF-IoD protocol, providing hardware-level resistance against physical capture. However, considering that the IoD emphasizes extreme lightweightness and specific hardware protection, the VANETs scenario exhibits stronger decentralized characteristics in its underlying architecture and has more complex requirements for conditional privacy protection. Therefore, designing an authentication architecture in VANETs that achieves lightweight authentication while balancing decentralized trust and strong privacy protection has become a critical research focus in the field of VANET security.

In summary, existing identity authentication methods in VANETs often face a difficult trade-off between computational efficiency and privacy protection strength. Some schemes incur high computation and communication overheads due to their reliance on complex bilinear pairing operations. Meanwhile, other schemes, while pursuing lightweight design, often face the problem of heavy pseudonym management burdens and exhibit certain limitations in achieving conditional privacy protection. To address the aforementioned issues, the paper proposes a privacy-preserving identity authentication and key agreement method for VANETs based on CL-PKC and NIZK. The method designs a dynamic anonymous identity generation mechanism combining a composite identity (CID) and Pedersen commitments. By storing static commitments containing identity information on the blockchain and generating one-time dynamic identities through re-randomization during each authentication, the scheme solves the certificate management issues and massive pseudonym maintenance overheads of traditional PKI. Consequently, it achieves the immutability of authentication metadata and strong anonymity. Regarding the authentication process, the proposed method integrates private key validity verification with dynamic identity verification. Vehicles only need to construct a single NIZK based on the Schnorr protocol to simultaneously complete the dual verification and securely negotiate a shared session key. This not only eliminates the key escrow risk of the system but also ensures the concealment of the vehicle’s real identity and the forward secrecy of communications.

## 3. Foundations and security objectives

### 3.1 Foundational models

#### 3.1.1 Elliptic curve cryptography.

ECC is a public-key cryptosystem based on elliptic curve theory that finds wide applications across various security domains. Its security relies on the computational hardness of the elliptic curve discrete logarithm problem, offering strong security with relatively short key lengths [28,29]. Let ${\text{F}}_{p}$ be a finite field where p is a large prime. An elliptic curve E defined over ${\text{F}}_{p}$ can be represented by the Weierstrass equation:


$$\begin{array}{c} y^{\text{2}}=x^{\text{3}}+ax+b \end{array}$$ (1)

Where $a,b\in {\text{F}}_{p}$. The cyclic group G of order k includes the point at infinity O (serving as the identity element) and a generator P.

1. **Elliptic Curve Discrete Logarithm Problem (ECDLP):** Given two points P and Q in the elliptic curve group G such that:

$$\begin{array}{c} Q=s\cdot \text{P} \end{array}$$ (2)

Where $s\in {\text{Z}}_{q}^{*}$. The computational hardness of ECDLP refers to the computational infeasibility for any probabilistic polynomial-time (PPT) adversary to derive the scalar s from the known points P and Q.

2. **Computational Diffie-Hellman Problem (CDHP):** Given a tuple (G,a·G,b·G), where $a,b\in {\text{Z}}_{q}^{*}$ are unknown randomly chosen scalars. The computational hardness of CDHP refers to the computational infeasibility for any PPT adversary to compute the point (ab)·G.

The security of elliptic curve cryptosystems depends on the computational hardness of the ECDLP and the CDHP. The security of the proposed method is precisely based on the assumed hardness of these two problems.

#### 3.1.2 Non-interactive zero-knowledge proof.

NIZK allows a prover to convince a verifier of a statement’s truthfulness without any interaction and without revealing any useful information. The core idea is to replace the verifier’s random challenges in interactive protocols with a public random oracle or a common reference string (CRS). An NIZK protocol for proving private key ownership can be defined as:


$$\begin{array}{c} \text{NIZK}\{sk:PK=sk\cdot G\} \end{array}$$ (3)

The prover P possesses the private key sk corresponding to the public key PK, satisfying PK=sk·G, where G is a fixed generator of an elliptic curve group. The standard NIZK protocol construction comprises a proof generation algorithm and a verification algorithm:

1. **Proof Generation Algorithm.** Prove(G,PK,sk)→π: Given generator G, public key PK, and private key sk, the algorithm generates a zero-knowledge proof π as follows:
  **Step 1:** Randomly select a temporary random number $r\in {\text{Z}}_{q}^{*}$ and compute the commitment R=r·G.
  **Step 2:** Generate a challenge c←H(R∥PK) by querying the random oracle H, then compute the response z=r+c·sk.
  **Step 3:** Output the proof tuple π=(R,z).
2. **Verification Algorithm.** Verify(G,PK,π)→b∈{0,1}: Given generator G, public key PK, and proof π=(R,z), the algorithm verifies the proof as follows:
  **Step 1:** Recompute the challenge c←H(R∥PK).
  **Step 2:** Verify whether z·G=R+c·PK holds. If true, output b=1 (accept), otherwise output b=0 (reject).

The protocol satisfies completeness, zero-knowledge, and knowledge soundness in the random oracle model. This construction enables the prover to efficiently generate a standalone and publicly verifiable proof, while the verifier only requires public parameters and the proof for verification. The entire process is non-interactive, ensuring private key confidentiality while enabling secure and efficient proof transmission.

#### 3.1.3 Certificateless public key cryptography.

CL-PKC combines the advantages of traditional PKI and IBC by employing a dual-key mechanism that prevents the key generation center from accessing users’ complete private keys. The CL-PKC framework comprises five algorithms: Setup, PartialKeyGen, UserKeyGen, SetPrivateKey, and SetPublicKey, detailed as follows:

1. **System Setup.** $Setup({\text{1}}^{\lambda })\to (s,P_{\text{pub}})$: Takes a security parameter λ as input. The PKG generates the master key pair, where the master private key $s\in {\text{Z}}_{q}^{*}$, the master public key $P_{\text{pub}}=s\cdot G$, and the system parameters are published.
2. **Partial Private Key Generation.** $PartialKeyGen(s,ID)\to D_{ID}$: The PKG uses the master private key s and user identity ID to generate the partial private key $D_{ID}=s\cdot Q_{ID}$, which is distributed to the user via a secure channel.
3. **User Key Generation.** $UserKeyGen({\text{1}}^{\lambda })\to (x_{ID},X_{ID})$: The user generates a secret value $x_{ID}\in {\text{Z}}_{q}^{*}$ and computes the corresponding public key component $X_{ID}=x_{ID}\cdot G$.
4. **Private Key Generation.** $SetPrivateKey(D_{ID},x_{ID})\to {sk}_{ID}$: The user combines the partial private key $D_{ID}$ with the secret value $x_{ID}$ to generate the complete private key $sk_{ID}$.
5. **Public Key Generation.** $SetPublicKey(Q_{ID},X_{ID})\to {pk}_{ID}$: The user combines the identity-based public key $Q_{ID}=H(ID)$ with the public key component $X_{ID}$ to generate the complete public key $pk_{ID}$.

This approach effectively eliminates key escrow risks by distributing private key generation between users and the PKG, while maintaining the convenience of identity-based cryptography.

### 3.2 System model

To address the issues of identity authentication and privacy protection in VANETs, the paper designs an anonymous identity authentication and key agreement model based on CL-PKC and the Schnorr protocol. The model employs a hierarchical collaborative architecture, consisting of four core entities: PKG, RSUs, On-Board Units (OBUs), and the blockchain platform. The model is illustrated in Fig 1.

**Fig 1:**
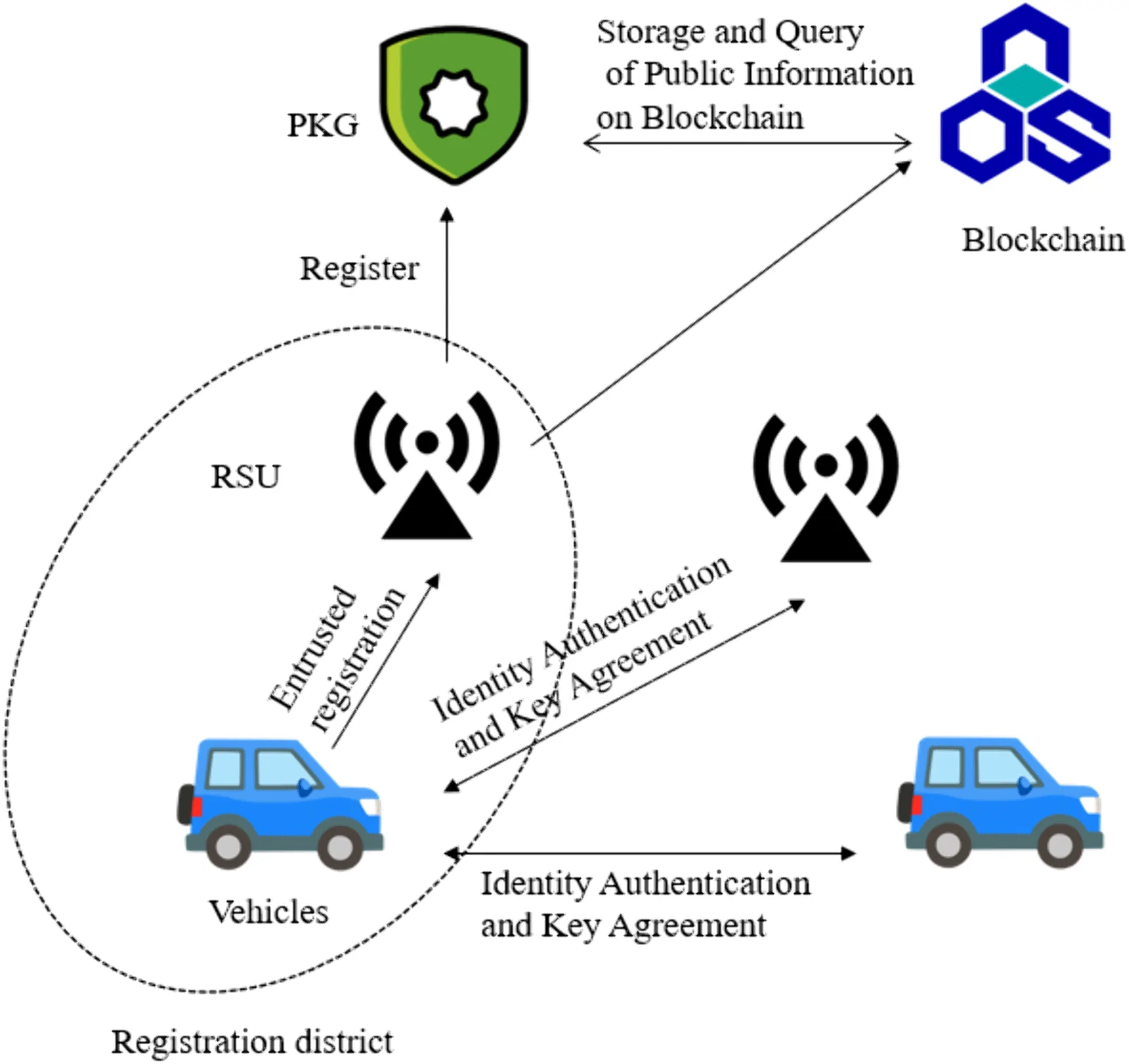
System model diagram. This figure was independently created by the authors to illustrate a standard VANET architecture. The car, RSU, PKG, and blockchain icons used in this diagram are sourced from the Noto Emojis collection by googlefonts under the Apache License 2.0.

1. **PKG:** As a trusted third-party entity, the PKG is responsible for generating the system’s global parameters and master key pair. The PKG performs system initialization, entity identity verification, and the distribution of partial private keys. Through CL-PKC, the PKG only participates in generating partial private key components for the vehicles, thereby eliminating the risk of key escrow.
2. **RSUs:** Roadside units deployed on both sides of the road act as distributed communication facilities and serve as verifiers during the authentication process. It is responsible for receiving and verifying anonymous authentication requests sent by vehicles and confirming the legitimacy of vehicle identities through the execution of NIZK algorithms.
3. **Vehicle:** Vehicles are equipped with OBUs to collect and broadcast information such as location, speed, and traffic conditions. Since vehicles require frequent authentication while driving at high speeds and OBUs have relatively limited computational resources, the OBU is responsible for constructing zero-knowledge proofs using lightweight algorithms to authenticate identities with RSUs without disclosing the real identity.
4. **Blockchain:** As a distributed ledger, the blockchain provides tamper-proof storage services. Given that VANETs involve multiple parties, including traffic management and operators, the use of blockchain, rather than traditional distributed databases, ensures that identity registration information and system parameters are not illegally altered under multi-party management. The proposed method is deployed on a consortium blockchain based on the PBFT consensus mechanism. The blockchain is responsible for recording the static commitment values (Commit) generated during vehicle registration and the Certificate Revocation List (CRL), ensuring that all RSU nodes can access a unified and tamper-proof verification basis.

### 3.3 Trust assumptions and threat model

#### 3.3.1 Trust assumptions.

The trust levels of different entities in the system are assumed as follows:

1. **PKG**: It is assumed to be a fully trusted entity. The PKG is responsible for system initialization and the generation of partial keys. It strictly safeguards the system’s master private key and will never conspire with any adversaries or entities.
2. **RSUs**: They are assumed to be honest-but-curious (semi-trusted) entities. RSUs strictly follow the protocol for vehicle authentication, data forwarding, and revocation tracking. However, they are curious and may attempt to infer the real identity and travel trajectory of vehicles from the communication data they collect. Additionally, since RSUs are deployed in open environments, it is assumed that a very small number of RSUs may be compromised by adversaries, either physically or via network attacks, leading to potential leakage of their private keys.
3. **Vehicles**: They are assumed to be untrusted entities. Vehicles may be fully controlled by adversaries, and malicious vehicles may attempt to forge legitimate identities, steal other session keys, or launch replay attacks.
4. **Blockchain**: It is assumed to be a fully trusted distributed public ledger with tamper-proof and single-point failure resistance capabilities. Based on the consensus mechanism, any data written to the blockchain (such as public parameters, vehicle identity commitments, and the CRL) is considered to be authentic and tamper-proof.

#### 3.3.2 Threat model.

The security analysis of this solution is based on the widely adopted Dolev-Yao (DY) threat model and the Canetti-Krawczyk (CK) adversary model for key agreement. The adversary A, assumed to be a PPT entity, possesses the following capabilities:

1. The adversary has full control over the public wireless communication channels between RSUs, as well as between RSUs and the PKG. The adversary can eavesdrop, intercept, tamper with, replay, or inject any messages transmitted over the public channels (such as authentication requests, Req).
2. The adversary can launch physical or network attacks to compromise some legitimate vehicles and extract their privately stored keys. The adversary may even compromise a few RSUs to gain access to their private keys and attempt to conspire to compromise the system’s security or privacy.
3. The adversary is unable to break the underlying standard cryptographic problems, such as the ECDLP and the CDHP, within polynomial time, nor can it perform pre-image or collision attacks on secure hash functions.

### 3.4 Security objectives

In VANETs, the highly dynamic and open nature of vehicular communications introduces significant challenges to identity authentication and key agreement processes. Challenges such as complex certificate management, key escrow risks, and susceptibility to identity privacy leaks hinder meeting the resource-constrained and high-real-time requirements of VANETs. The paper proposes an anonymous authentication and key agreement method based on CL-PKC and NIZK. The proposed method avoids key escrow while achieving vehicle identity anonymity and communication unlinkability, ensuring the forward secrecy of session keys and resistance to common attacks.

1. **Identity Anonymity and Unlinkability.** The method ensures that a vehicle’s real identity remains undisclosed during all communication processes. Adversaries cannot obtain a vehicle’s true identity through active attacks (e.g., forged authentication requests) or passive attacks (e.g., eavesdropping). Furthermore, adversaries cannot link different sessions to the same vehicle by analyzing multi-session communication data, thus effectively preventing the tracking and reconstruction of user behavior patterns.
2. **Strong Forward Secrecy.** The method provides strong forward secrecy, ensuring that even if adversaries compromise long-term keys, they cannot recover previous session keys, thus protecting the confidentiality of historical communications.
3. **Resistance to Common Attacks.** The method effectively resists various attacks common in VANETs, including replay, man-in-the-middle, collusion, spoofing, and key-leak impersonation attacks. It ensures the integrity and reliability of the authentication and key agreement process.

## 4. Research content and methodology

### 4.1 Proposed authentication method

The paper proposes a VANETs anonymous identity authentication and key agreement method based on CL-PKC and NIZK, which ensures vehicle identity privacy protection, anonymous identity authentication, and forward-secure key agreement. The method is divided into six stages: system initialization, RSU registration, vehicle registration, anonymous identity authentication, Key agreement, and tracking and revocation. The system flowchart is shown in Fig 2.

**Fig 2:**
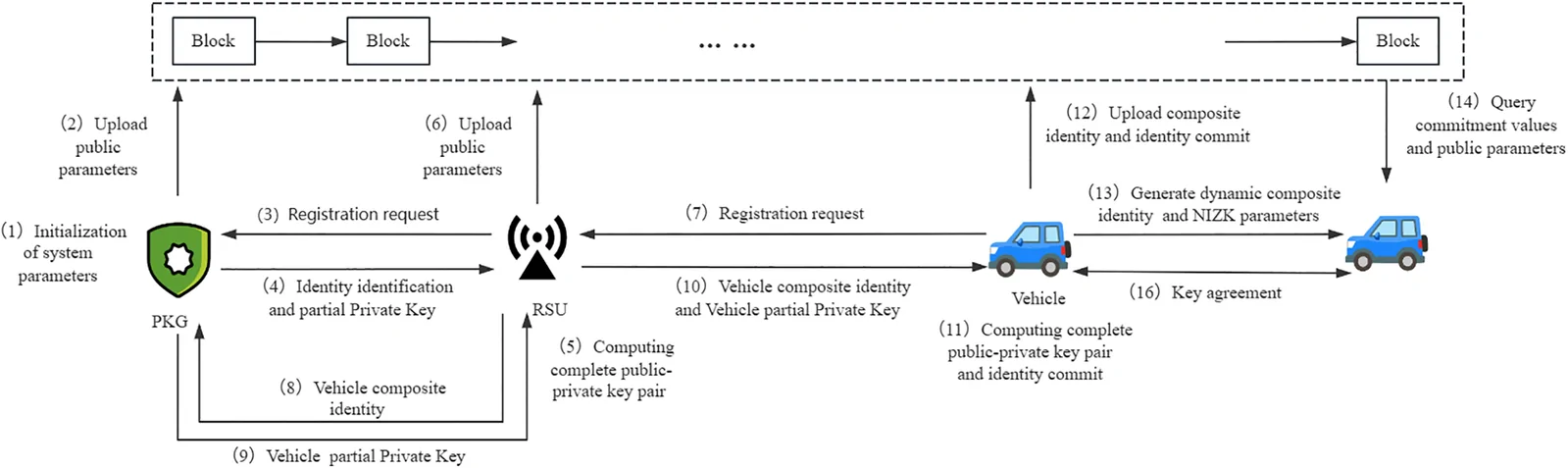
System flowchart. This flowchart was independently generated by the authors to illustrate the proposed methodology. The icons (such as the car, RSU, and PKG) used in this diagram are sourced from the Noto Emojis collection by googlefonts under the Apache License 2.0.

In the proposed method, the blockchain serves as the underlying distributed trusted storage medium and auditing platform. It does not directly participate in complex cryptographic computations but is responsible for securely storing the system’s public parameters (Params), the public key components of RSUs, vehicle static commitment values (Commit), and the Certificate Revocation List (CRL). This design effectively mitigates the single-point failure risk inherent in traditional centralized architectures, allowing RSUs to efficiently and reliably query the legitimate registration credentials and other public parameters of vehicles during identity verification, and to quickly perform consistent revocation across the entire network in the event of vehicle violations.

The design of the composite identity (CID) is the foundation for achieving anonymity and unlinkability in the method. The static composite identity $CID_{i}$ binds the vehicle’s real identity, the RSU area identifier, and a timestamp, ensuring the uniqueness of the vehicle registration while isolating its real identity. Meanwhile, the $CID_{new}$ dynamically generated during a single authentication process re-randomizes the static identity by introducing a random number. This makes the authentication data generated by the same vehicle across multiple requests completely unlinkable, thereby effectively protecting the vehicle’s trajectory privacy and identity privacy. The main symbols involved in the paper and their corresponding descriptions are shown in Table 1.

**Table 1:** List of symbols.

Symbol	Description
$P_{pub}$	System’s public key
sk	System’s private key
$H_{\text{1}},H_{\text{2}},H_{\text{3}},H_{\text{4}}$	Collision-resistant hash functions
${VID}_{i}$	Unique identity identifier of vehicle $V_{i}$
${RID}_{j}$	Unique identity identifier $RSU_{j}$
${CID}_{i}$	Composite identity identifier of vehicle $V_{i}$
${CID}_{new}$	Dynamic composite identity in a single authentication
$r{sk}_{j}$	Complete private key of $RSU_{j}$
$R{PK}_{j}$	Complete public key of $RSU_{j}$
${vsk}_{i}$	Complete private key of vehicle $V_{i}$
${VPK}_{i}$	Complete public key of vehicle $V_{i}$
Commit	Vehicle identity commitment value

#### 4.1.1 System initialization phase.

The system initialization phase is executed by the PKG, which establishes the foundational cryptographic parameters for the entire system and provides a secure basis for subsequent phases. The specific steps are as follows:

**Step 1:** The PKG selects secure elliptic curve parameters $\left(E/{\text{F}}_{p},G,q\right)$ for all subsequent operations, including key generation, signature verification, and key agreement. The PKG also selects another generator H on the elliptic curve. Here, p is a large prime, G is a base point on the elliptic curve, and q is the prime order of G. And $E/{\text{F}}_{p}$ is an elliptic curve defined over the finite field ${\text{F}}_{p}$, with the equation:

$$\begin{array}{c} y^{\text{2}}=x^{\text{3}}+ax+b \end{array}$$ (1)

**Step 2:** The PKG selects four cryptographic hash functions $H_{\text{1}},H_{\text{2}},H_{\text{3}},H_{\text{4}}$, defined as follows:
  ① $H_{\text{1}}:\{\text{0},\text{1}{\}}^{*}\to Z_{q}^{*}$, used to map identity information.
  ② $H_{\text{2}}:\{\text{0},\text{1}{\}}^{*}\to Z_{q}^{*}$, generating random challenges and key derivation parameters.
  ③ $H_{\text{3}}:\{\text{0},\text{1}{\}}^{*}\to \{\text{0},\text{1}{\}}^{\lambda }$, generating symmetric keys.
  ④ $H_{\text{4}}:\{\text{0},\text{1}{\}}^{*}\to \{\text{0},\text{1}{\}}^{\lambda }$, used for message authentication code generation and integrity verification.
**Step 3:** The PKG randomly selects the master private key $s\in Z_{q}^{*}$ and computes the master public key $P_{pub}=s\cdot G$. The system’s public parameters are:

$$\begin{array}{c} Params=\{p,q,E/{\text{F}}_{p},G,H,Ppub,H_{\text{1}},H_{\text{2}},H_{\text{3}},H_{\text{4}}\} \end{array}$$ (4)

The PKG then uploads Params to the blockchain and stores s securely.

The system initialization phase completes the establishment of the cryptographic foundation, providing essential parameter infrastructure for subsequent entity registration and secure communication.

#### 4.1.2 RSU registration phase.

The RSU registration phase is executed by the PKG to enable RSUs to obtain the cryptographic parameters required for subsequent authentication and key agreement processes, establishing legitimate system identities. The specific process, illustrated in Fig 3, is detailed below:

**Fig 3:**
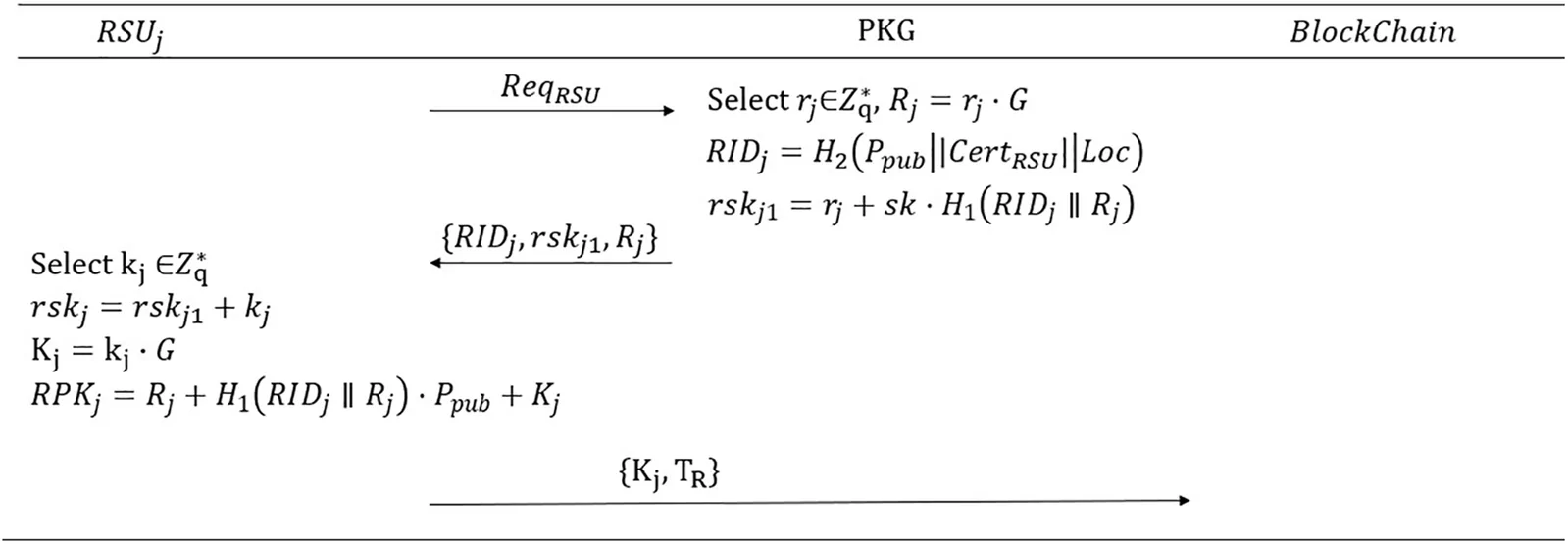
RSU registration phase.

**Step 1:** ${RSU}_{j}$ sends a registration request ${Req}_{RSU}$ to the PKG:

$$\begin{array}{c} {Req}_{RSU}=({Cert}_{RSU},\text{Loc},\sigma_{CA}) \end{array}$$ (5)

Where ${\text{Cert}}_{RSU}$ is the valid digital certificate of ${RSU}_{j}$, Loc is its geographic location information, and $\sigma_{CA}$ is the Certificate Authority signature.

**Step 2:** Upon receiving the request, the PKG validates $\sigma_{CA}$ and checks whether ${Cert}_{RSU}$ is valid and not revoked. It then generates the unique identifier ${RID}_{j}$:

$$\begin{array}{c} {RID}_{j}=H_{\text{2}}\left(P_{pub}\left|\left|{Cert}_{RSU}\right|\right|Loc\right) \end{array}$$ (6)

The PKG randomly selects $r_{j}\in Z_{q}^{*}$ and computes $R_{j}=r_{j}\cdot G$. Then, it computes the partial private key component $rsk_{j\text{1}}$ for $RSU_{j}$ using the system master private key, and transmits $\{RID_{j},rsk_{j\text{1}},R_{j}\}$ to $RSU_{j}$ via a secure channel.


$$\begin{array}{c} sk_{j\text{1}}=r_{j}+sk\cdot H_{\text{1}}\left(RID_{j}∥R_{j}\right) \end{array}$$ (7)

**Step 3:**${RSU}_{j}$ randomly selects $k_{i}\in {\text{Z}}_{q}^{*}$ as another private key component and computes the full private key $r{sk}_{j}$ and full public key $R{PK}_{j}$:

$$\begin{array}{c} {sk}_{j}=r{sk}_{j\text{1}}+k_{j} \end{array}$$ (8)

$$\begin{array}{c} RPK_{j}=R_{j}+H_{\text{1}}\left(RID_{j}∥R_{j}\right)\cdot P_{pub}+K_{j} \end{array}$$ (9)

It submits $\left\{K_{i},T_{R},R_{j}\right\}$ to the blockchain, where $K_{i}=k_{i}\cdot G$ represents the public key component of ${RSU}_{j}$, and $T_{R}$ denotes the registration timestamp.

This phase achieves secure registration and key distribution for RSUs within the system. The partial private key mechanism mitigates key escrow risks while establishing a trusted identity foundation for RSUs to participate in subsequent vehicle authentication processes.

#### 4.1.3 Vehicle registration phase.

The goal of the vehicle registration phase is to allow legitimate vehicles $V_{i}$ to complete anonymous registration with the assistance of the RSU in their respective regions, generating the private key bound to their identity and the static commitment values required for the zero-knowledge proof. After entering the designated area, the vehicle submits a registration request to the RSU in that area, which includes the vehicle’s digital certificate ${Cert}_{V}$ and a nonce. Upon verifying the validity of the vehicle’s certificate, the RSU will execute the following registration process. The specific flow is illustrated in Fig 4, with the detailed procedure described below:

**Fig 4:**
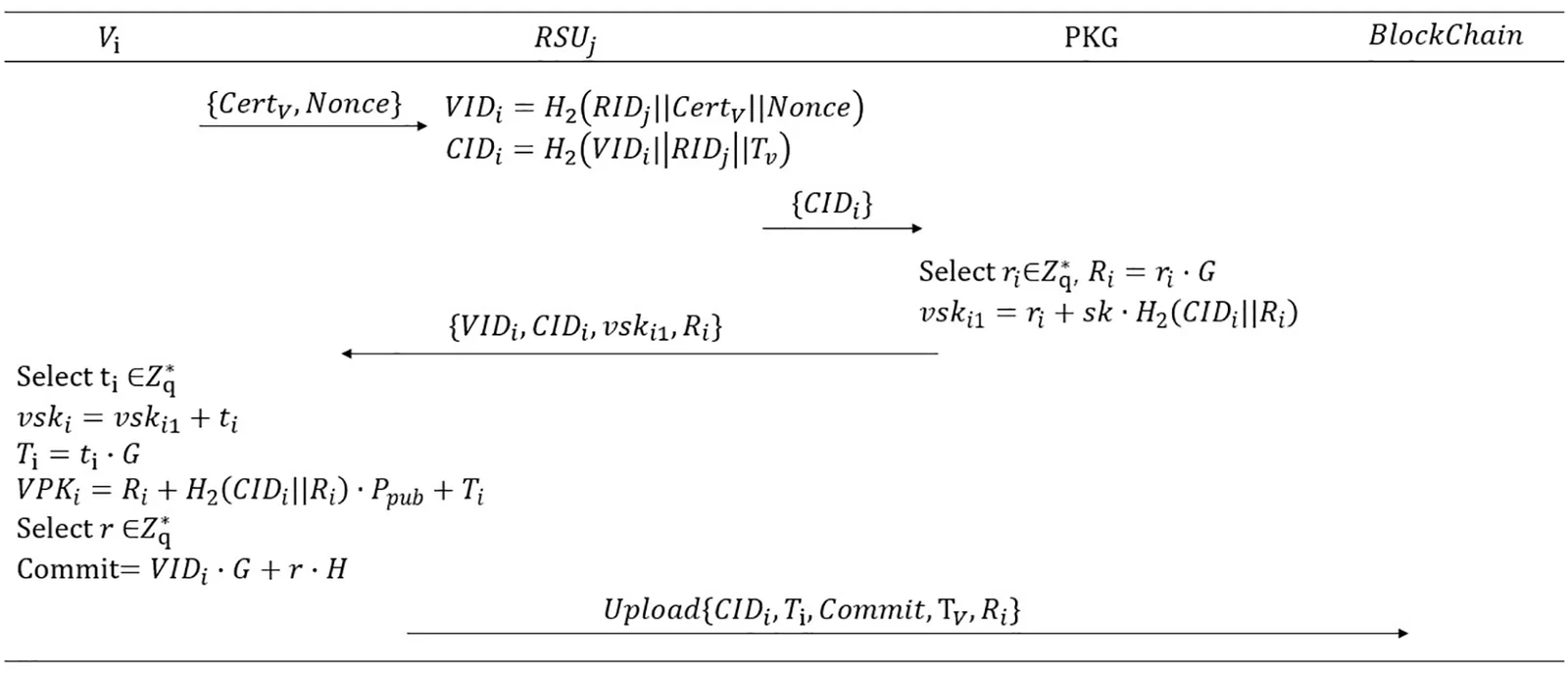
Vehicle registration phase.

**Step 1:** The vehicle submits its identity information and registration request to ${RSU}_{j}$. After successful verification, ${RSU}_{j}$ generates a unique vehicle identifier ${VID}_{i}=H_{\text{2}}\left({RID}_{j}|\left|{Cert}_{V}\right||Nonce\right)$. Combined with its own identifier ${RSU}_{j}$, it then generates a composite identifier ${CID}_{i}$:

$$\begin{array}{c} {CID}_{i}=H_{\text{2}}\left({VID}_{i}|\left|{RID}_{j}\right||T_{v}\right) \end{array}$$ (10)

Where ${\text{VID}}_{\text{i}}$ is the vehicle’s unique identity identifier, ${RID}_{j}$ is the identity identifier of the registering ${RSU}_{j}$ and $T_{V}$ is the vehicle’s registration timestamp.

**Step 2:** $RSU_{j}$ sends the composite identity $CID_{i}$ to the PKG. After verifying the identity of $RSU_{j}$, the PKG randomly selects a secret value $r_{i}\in Z_{q}^{*}$ and computes $R_{i}=r_{i}\cdot G$. Subsequently, it generates the partial private key $vsk_{i\text{1}}$ for the vehicle and securely transmits $\{CID_{i},vsk_{i\text{1}},R_{i}\}$ to the vehicle via $RSU_{j}$.

$$\begin{array}{c} {vsk}_{i\text{1}}=r_{i}+sk\cdot H_{\text{2}}\left({CID}_{i}||R_{i}\right) \end{array}$$ (11)

**Step 3:** The vehicle randomly selects $t_{i}\in {\text{Z}}_{q}^{*}$ as a private key component and computes the full private key ${vsk}_{i}$ and full public key ${VPK}_{i}$:

$$\begin{array}{c} {vsk}_{i}={vsk}_{i\text{1}}+t_{i} \end{array}$$ (12)

$$\begin{array}{c} {VPK}_{i}=R_{i}+H_{\text{2}}\left({CID}_{i}||R_{i}\right)\cdot P_{pub}+T_{i} \end{array}$$ (13)

Where $T_{i}=t_{i}\cdot G$ is the vehicle’s public key component.

**Step 4:** The vehicle randomly selects $r\in {\text{Z}}_{q}^{*}$ and computes the commitment value Commit:

$$\begin{array}{c} \text{Commit}={VID}_{i}\cdot G+r\cdot H \end{array}$$ (14)

Where H is another generator of the elliptic curve. The blockchain stores the vehicle registration record ${\text{CID}}_{i},T_{i},\text{Commit},T_{V},R_{i}$.

In this phase, through the use of composite identity and Pedersen commitment techniques, the vehicle successfully acquires the communication key and completes the on-chain notarization. Throughout the process, the vehicle’s real identity is not exposed on the public network or blockchain, thus laying the foundation for achieving anonymous identity authentication in the authentication phase.

#### 4.1.4 Anonymous identity authentication phase.

In the anonymous identity authentication phase, before initiating communication, the vehicle must prove its legitimacy to the target $RSU_{j}$. The phase first uses re-randomization to generate the dynamic composite identity for the current authentication, and then applies NIZK based on the Schnorr protocol. This allows the vehicle to prove possession of the private key corresponding to the on-chain Commit without exposing its real identity, thus achieving secure and unlinkable authentication. The specific process, illustrated in Fig 5, is described below:

**Fig 5:**
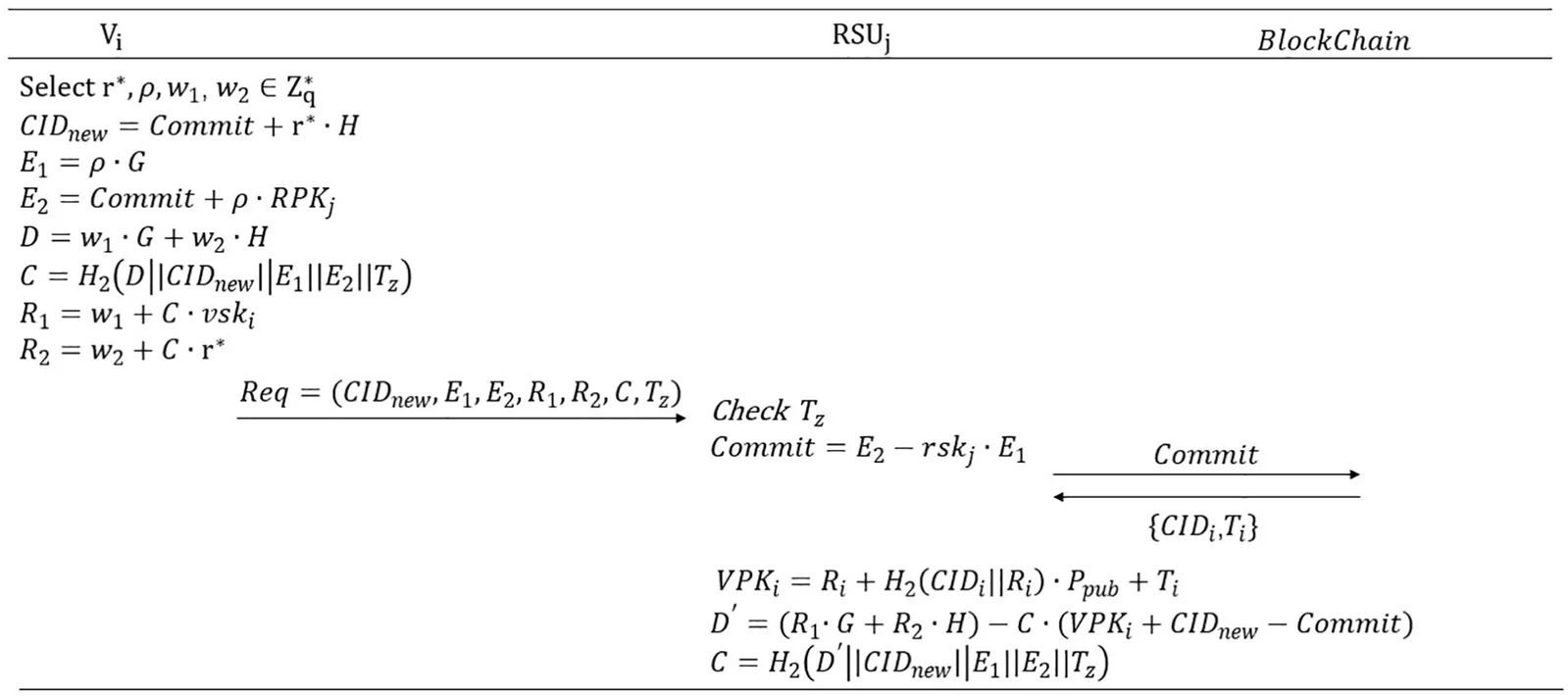
Anonymous identity authentication phase.

**Step 1:** Vehicle $V_{i}$ first selects a re-randomization factor $r^{*}\in {\text{Z}}_{q}^{*}$, and based on the static Commit, generates the dynamic composite identity for the current communication ${CID}_{new}$:

$$\begin{array}{c} {CID}_{new}=Commit+{\text{r}}^{*}\cdot H \end{array}$$ (15)

To achieve unlinkability and hide the static Commit, the vehicle generates a temporary random number $\rho \in {\text{Z}}_{q}^{*}$, and uses the public key of the verifier $RSU_{j}$, $R{PK}_{j}$, to blind the Commit by encrypting it:


$$\begin{array}{c} E_{\text{1}}=\rho \cdot G \end{array}$$ (16)

$$\begin{array}{c} E_{\text{2}}=Commit+\rho \cdot R{PK}_{j} \end{array}$$ (17)

Next, vehicle $V_{i}$ selects random numbers $w_{\text{1}},w_{\text{2}}\in {\text{Z}}_{q}^{*}$, and computes the obfuscation value D, the challenge value C, and the response values $R_{\text{1}}$ and $R_{\text{2}}$ for the NIZK:


$$\begin{array}{c} D=w_{\text{1}}\cdot G+w_{\text{2}}\cdot H \end{array}$$ (18)

$$\begin{array}{c} C=H_{\text{2}}\left(D\left|\left|{CID}_{new}\right|\right|E_{\text{1}}|\left|E_{\text{2}}\right||T_{z}\right) \end{array}$$ (19)

$$\begin{array}{c} R_{\text{1}}=w_{\text{1}}+C\cdot {vsk}_{i} \end{array}$$ (20)

$$\begin{array}{c} R_{\text{2}}=w_{\text{2}}+C\cdot {\text{r}}^{*} \end{array}$$ (21)

**Step 2:** Vehicle $V_{i}$ sends the authentication request Req to the verifier:

$$\begin{array}{c} Req=({CID}_{new},E_{\text{1}},E_{\text{2}},R_{\text{1}},R_{\text{2}},C,T_{z}) \end{array}$$ (22)

where $T_{z}$ is the identity authentication timestamp.

**Step 3:** Upon receiving the request Req, the verifier $RSU_{j}$ first checks whether the timestamp $T_{z}$ falls within the valid time window to prevent replay attacks. It then decrypts and recovers the vehicle’s static commitment value Commit using its private key:

$$\begin{array}{c} Commit=E_{\text{2}}-r{sk}_{j}\cdot E_{\text{1}} \end{array}$$ (23)

The verifier $RSU_{j}$ then uses the recovered Commit as an index to query the blockchain state. First, it ensures that the Commit exists in the legitimate registration list and has not been listed in the revocation list. Next, it retrieves the corresponding vehicle’s ${CID}_{i}$ and $T_{i}$, and reconstructs the vehicle’s full public key:


$$\begin{array}{c} {VPK}_{i}=R_{i}+H_{\text{2}}\left({CID}_{i}||R_{i}\right)\cdot P_{pub}+T_{i} \end{array}$$ (13)

**Step 4:** $RSU_{j}$ uses the received NIZK parameters to derive the proof verification value $D^{\prime }$:

$$\begin{array}{c} D^{\prime }={(R}_{\text{1}}\cdot G+R_{\text{2}}\cdot H)-C\cdot ({VPK}_{i}+{CID}_{new}-Commit) \end{array}$$ (24)

It then verifies whether the following challenge equation holds:


$$\begin{array}{c} {C=H}_{\text{2}}\left(D^{\prime }\left|\left|{CID}_{new}\right|\right|E_{\text{1}}|\left|E_{\text{2}}\right||T_{z}\right) \end{array}$$ (25)

After all verifications pass, the authentication is successful, and both parties enter the key agreement phase. Any failure in the verification steps will result in authentication failure, and the verifier must immediately terminate the protocol and reject the connection request.

In this phase, efficient identity verification is achieved through NIZK, which protects the vehicle’s identity privacy while ensuring the legitimacy of the communicating entities.

#### 4.1.5 Key agreement phase.

The key agreement and secure communication phase establishes a secure session key after successful authentication, ensuring confidentiality and integrity for subsequent communications. The process, illustrated in Fig 6, is detailed below:

**Fig 6:**
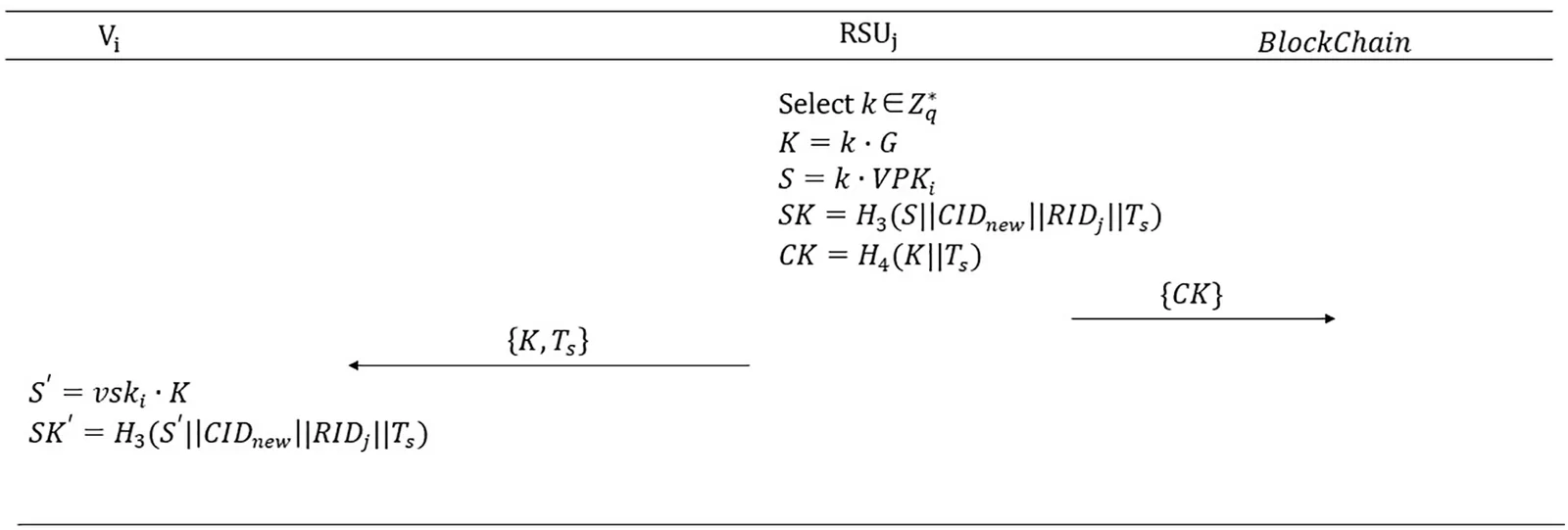
Key agreement phase.

**Step 1:** After confirming the vehicle’s legitimate identity, the verifier randomly selects a session key seed parameter $k\in {\text{Z}}_{q}^{*}$, computes K=k·G, and sends $\left\{K,T_{s}\right\}$ to the vehicle. The RSU calculates the shared secret $S=k\cdot {VPK}_{i}$ and derives the session key as:

$$\begin{array}{c} SK=H_{\text{3}}(S|\left|{CID}_{new}\right||{RID}_{j}||T_{s}) \end{array}$$ (26)

Simultaneously, the commitment value $CK=H_{\text{4}}(K||T_{s})$ is stored on the blockchain for auditing.

**Step 2:** Upon receiving K, the vehicle uses its private key to compute the shared secret $S^{\prime }={vsk}_{i}\cdot K$ and derives the identical session key:

$$\begin{array}{c} {SK}^{\prime }=H_{\text{3}}(S^{\prime }|\left|{CID}_{new}\right||{RID}_{j}||T_{s}) \end{array}$$ (27)

All subsequent communications are encrypted and decrypted using this session key. When sending data to the RSU, the vehicle encrypts the plaintext with the session key and appends a Message Authentication Code (MAC) to ensure data integrity and authenticity. Upon receipt, the RSU first validates the MAC before decrypting the ciphertext with the session key. Similarly, the RSU follows the same procedure when transmitting data to the vehicle. After each communication session, the session key is immediately destroyed. A new key must be negotiated for subsequent communications to ensure forward secrecy.

#### 4.1.6 Tracking and revocation phase.

In the VANETs environment, when a vehicle exhibits malicious behavior, such as sending false alerts or launching denial-of-service attacks, the system must be capable of tracing its identity and revoking its legitimate communication credentials. In this phase, collaboration between the RSU and the PKG enables the tracing of malicious vehicles and the revocation of their credentials. The specific steps are as follows:

**Step 1:** When $RSU_{j}$ detects an anomaly or receives a violation report from another vehicle, it extracts the malicious communication’s request packet $Req=({CID}_{new},E_{\text{1}},E_{\text{2}},R_{\text{1}},R_{\text{2}},C,T_{z})$ along with other relevant violation evidence. Subsequently, $RSU_{j}$ uses its private key $r{sk}_{j}$ to recover the vehicle’s real static identity commitment value:

$$\begin{array}{c} Commit=E_{\text{2}}-r{sk}_{j}\cdot E_{\text{1}} \end{array}$$ (23)

Then, $RSU_{j}$ sends the recovered Commit along with the violation evidence to the PKG via a secure channel to initiate the tracing request.

**Step 2:** After receiving the tracing request and validating the evidence, the PKG performs the following query operations: First, the PKG queries the Commit in the blockchain’s public ledger to retrieve the corresponding vehicle registration record $\{{CID}_{i},T_{i},Commit,T_{v}$}, from which it extracts the composite identity identifier ${CID}_{i}$. Then, based on the retrieved ${CID}_{i}$, the PKG queries its internal registration database’s mapping table to obtain the real digital certificate ${Cert}_{V}$ associated with the vehicle’s registration, thereby locking onto the malicious vehicle’s true identity.
**Step 3:** Once the identity is locked, the PKG writes the vehicle’s Commit and $CID_{i}$ into the blockchain’s global Certificate Revocation List (CRL). When processing subsequent concurrent requests, RSUs across the network can automatically reject the vehicle’s authentication requests by querying the on-chain status.

Through the protocol interactions in the above six phases, the proposed authentication and key agreement method constructs a complete conditional privacy-preserving architecture. The method integrates CL-PKC, NIZK, and blockchain technologies. It utilizes the blockchain to replace traditional centralized databases, serving as an immutable distributed ledger to store the vehicle’s static commitments (Commit) and the CRL, thereby effectively avoiding single points of failure. Meanwhile, by combining the CL-PKC mechanism, the private key is jointly generated by the PKG and the entity, which avoids the certificate management overhead and key escrow risks in the traditional PKI architecture. Regarding privacy protection and unlinkability, the method designs an architecture combining a composite identity (CID) and Pedersen commitments. Each time a vehicle initiates an authentication request, it introduces a random number to re-randomize the static commitment, generating a dynamic composite identity $CID_{new}$. This makes the authentication credentials generated by the same vehicle at different times and under different RSU coverage areas cryptographically unlinkable, effectively protecting the vehicle’s trajectory privacy. In terms of authentication efficiency and identity tracking, the vehicle only needs to construct a NIZK based on the Schnorr protocol to complete the legitimacy proof without leaking private information, which effectively reduces computation and communication overheads. Furthermore, during normal communication, the vehicle’s identity remains anonymous to the RSU and external eavesdroppers. When malicious behavior is detected, the RSU and PKG collaborate to extract the vehicle’s real identity and complete the on-chain revocation. In summary, the proposed method effectively balances the computational efficiency and privacy protection requirements in VANETs authentication, realizing a decentralized and lightweight identity authentication method suitable for VANET environments.

## 5. Security analysis

This section primarily evaluates the security of the proposed scheme. Initially, a game-based formal security proof is employed to verify the security of the session key and the unforgeability of the authentication requests; subsequently, ProVerif automated verification is conducted to ensure the confidentiality and mutual authentication of the protocol; finally, an informal security analysis is presented to elaborate on the defense capabilities of the proposed scheme against various common attacks in VANET scenarios.

### 5.1 Formal security analysis

This section employs a game-based sequence of proofs under the random oracle model (ROM) to formally prove the semantic security of the session key for the proposed scheme in the real-or-random (ROR) model, alongside the existential unforgeability against adaptive chosen-message attacks (EUF-CMA security) of the anonymous authentication requests.

Let A be a PPT adversary, and the x-th and y-th instances of the communicating entities, vehicle $V_{i}$ and $RSU_{j}$, be denoted as $\Pi_{V}^{x}$ and $\Pi_{RSU}^{y}$, respectively. The adversary A can simulate its control over the network channels and physical devices by executing the following standard oracle queries:

$\text{Execute}\left(\Pi_{V}^{x},\Pi_{RSU}^{y}\right)$. This query simulates the passive eavesdropping capability of the adversary. A can obtain the message $Req=\left(CID_{new},E_{\text{1}},E_{\text{2}},R_{\text{1}},R_{\text{2}},C,T_{z}\right)$ and the response $\left(K,T_{s}\right)$ exchanged over the public channel.
$\text{Send}\left(\Pi^{\times },m\right)$: This query simulates the active attack capability of the adversary. A can send any forged or tampered message m to the instance $\Pi^{\times }$($\Pi_{V}^{x}\;or\;\Pi_{RSU}^{y}$) and obtain the operational response of the entity.
Hash(m). This query allows the adversary to obtain the random oracle hash value corresponding to the message m.
$\text{Corrupt}\left(\Pi_{V}^{x}\right)$. This query simulates a long-term key compromise attack. A can compromise the physical device of the vehicle to obtain its stored static private key or secret parameters.
$\text{Reveal}\left(\Pi^{\times }\right)$. If the instance has successfully established a session key, this query returns the real session key SK of the current session to A.
$\text{Test}\left(\Pi^{\times }\right)$. This query is used to evaluate the semantic indistinguishability of the key. The challenger C tosses an unbiased coin b∈{0,1}. If b=1, it returns the real session key SK. If b=0, it returns a random bit string of the same length as SK.

**Theorem 1:** If a PPT adversary A can break the semantic security of the session key for the proposed scheme in the ROR model with a non-negligible advantage ${\text{Adv}}_{A}^{\text{AKA}}$ within polynomial time, then there must exist a challenger C capable of breaking the ECDLP or the CDHP with a non-negligible advantage.

**Proof:** Given a sequence of games ${\text{Game}}_{k}$, for k=0,1,2,3,4, the adversary A attempts to break the security of the proposed scheme. Let $\text{Pr}\left[W_{k}\right]$ denote the probability that the adversary A successfully guesses the outcome in ${\text{Game}}_{k}$ within probabilistic polynomial time. Subsequently, the detailed instances of each game are discussed.

${𝐆𝐚𝐦𝐞}_{0}$**:** This initial game simulates the actual attack of the adversary against the proposed scheme under the ROR model. The challenger randomly tosses a coin b. By definition, the advantage of the adversary A in breaking the protocol is given by:


$$\begin{array}{c} {\text{Adv}}_{A}^{\text{AKA}}=\left|\text{2}\cdot \text{Pr}\left[W_{\text{0}}\right]-\text{1}\right| \end{array}$$ (28)

${𝐆𝐚𝐦𝐞}_{1}$**:** This game represents the passive attack of adversary A. Adversary A can execute an unbounded number of $\text{Execute}\left(\Pi_{V}^{x},\Pi_{RSU}^{y}\right)$ queries, thereby intercepting all interactive messages $\left\{CID_{new},E_{\text{1}},E_{\text{2}},R_{\text{1}},R_{\text{2}},C,K,T_{z},T_{s}\right\}$ transmitted over the channel. However, the final session key $SK=H_{\text{3}}\left(S∥CID_{new}∥RID_{j}∥T_{s}\right)$ is derived from the Diffie-Hellman shared secret $S=k\cdot VPK_{i}$. Since A cannot recover the ephemeral random scalar k or the vehicle’s private key $vsk_{i}$ from the public parameters, passive eavesdropping provides no effective advantage to A regarding the shared secret S. Consequently, the winning probability of the adversary remains completely identical to that in ${\text{Game}}_{\text{0}}$:


$$\begin{array}{c} \text{Pr}\left[W_{\text{1}}\right]=\text{Pr}\left[W_{\text{0}}\right] \end{array}$$ (29)

${𝐆𝐚𝐦𝐞}_{2}$**:** This game represents the active attacks executed by adversary A. The adversary is permitted to execute Send queries and Hash queries, attempting to deceive the entities by forging false messages or finding collisions in the hash functions. According to the birthday paradox, when the size of the hash space is |Hash| and $q_{h}$ hash queries and $q_{s}$ Send queries are executed, the probability of a hash collision or a successful match occurring is negligible. Therefore, it holds that:


$$\begin{array}{c} \left|\text{Pr}\left[W_{\text{2}}\right]-\text{Pr}\left[W_{\text{1}}\right]\right|\leq \frac{q_{h}^{\text{2}}+q_{s}^{\text{2}}}{\text{2}|\text{Hash}|} \end{array}$$ (30)

${𝐆𝐚𝐦𝐞}_{3}$: This game considers a stronger adversary capability, namely, the long-term key compromise attack. The adversary A can execute Corrupt queries to obtain the sensitive data stored in the vehicle’s local physical device. In the proposed scheme, the complete private key of the vehicle consists of two parts: $vsk_{i}=\left(vsk_{i\text{1}},t_{i}\right)$, where the partial private key $vsk_{i\text{1}}$ is generated by the PKG during the partial private key distribution phase, whereas $t_{i}$ is randomly and autonomously generated by the vehicle within its local physical secure domain and is never exposed externally. Furthermore, the system employs a non-interactive zero-knowledge proof (NIZK) to protect the vehicle’s authentication privacy, and the static commitment value Commit is blindly encrypted into $\left(E_{\text{1}},E_{\text{2}}\right)$ using the public key $RPK_{j}$ of the target RSU. Since the adversary cannot breach the underlying physical secure domain to acquire $t_{i}$ and cannot decrypt $\left(E_{\text{1}},E_{\text{2}}\right)$, the advantage gain in the adversary’s winning probability for this game is negligible. Let ${\text{Adv}}_{A}^{PUF}$ denote the advantage of the adversary in breaching the physical hardware secure domain; then, it holds that:


$$\begin{array}{c} \left|\text{Pr}\left[W_{\text{3}}\right]-\text{Pr}\left[W_{\text{2}}\right]\right|\leq {\text{Adv}}_{A}^{PUF} \end{array}$$ (31)

${𝐆𝐚𝐦𝐞}_{4}$**:** This game simulates the scenario where the adversary A launches an adaptive chosen-message attack to completely forge a fully valid anonymous authentication request message $Req^{*}=\left(CID_{new}^{*},E_{\text{1}}^{*},E_{\text{2}}^{*},R_{\text{1}}^{*},R_{\text{2}}^{*},C^{*},T_{z}^{*}\right)$ to impersonate a legitimate vehicle, thereby passing the verification of the RSU’s NIZK challenge equation. ${\text{Game}}_{\text{4}}$ reduces the deceptive behavior of the adversary to the solution of the ECDLP. Given an ECDLP instance P,Y=x·P, the goal of the challenger C is to solve for x. C sets the system master public key or a partial public key component of the target vehicle to Y. If the adversary A can successfully forge an authentication request satisfying the verification equation $C^{*}=H_{\text{2}}\left(D^{\prime }∥CID_{new}^{*}∥Commit^{*}∥E_{\text{2}}^{*}∥T_{z}^{*}\right)$ with a non-negligible advantage, then according to the Fiege-Fiat-Shamir Forking Lemma, C can replay the adversary A under identical initialization conditions and pseudo-random numbers, and execute a fork at the random oracle $H_{\text{2}}$ to return a different hash challenge value $C^{{*}^{\prime }}$ (where $C^{*}\text{≠}C^{{*}^{\prime }}$). By extracting the dual valid NIZK responses provided by A for the two forks:


$$\begin{array}{c} R_{\text{1}}^{*}=w_{\text{1}}+C^{*}\cdot vsk_{i}\;(\text{mod }q) \end{array}$$ (32)

$$\begin{array}{c} R_{\text{1}}^{{*}^{\prime }}=w_{\text{1}}+C^{{*}^{\prime }}\cdot vsk_{i}\;(\text{mod }q) \end{array}$$ (33)

The challenger C can directly cancel out the blinding random number $w_{\text{1}}$ by subtracting the two linear equations, thereby precisely extracting the underlying long-term private key of the vehicle:


$$\begin{array}{c} vsk_{i}=\left(R_{\text{1}}^{*}-R_{\text{1}}^{{*}^{\prime }}\right)\cdot {\left(C^{*}-C^{{*}^{\prime }}\right)}^{-\text{1}}\;(\text{mod }q) \end{array}$$ (34)

Since this private key is mathematically tightly bound to the discrete logarithm challenge instance Y, C can directly solve for the unknown x, thereby breaking the ECDLP instance. Similarly, recovering historical session keys requires solving the CDHP instance based on this foundation. Since the computational advantage of breaking the ECDLP and CDHP within polynomial time is negligible under standard cryptographic assumptions, it holds that:


$$\begin{array}{c} \left|\text{Pr}\left[W_{\text{4}}\right]-\text{Pr}\left[W_{\text{3}}\right]\right|\leq {\text{Adv}}_{A}^{ECDLP} \end{array}$$ (35)

In ${\text{Game}}_{\text{4}}$, since the underlying hard mathematical problems are widely acknowledged as intractable, the adversary A cannot obtain any semantic information regarding the real session key, and its execution of the Test query can only rely on pure random guessing; thus:


$$\begin{array}{c} \text{Pr}\left[W_{\text{4}}\right]=\frac{\text{1}}{\text{2}} \end{array}$$ (36)

Combining Equations (28)–(36) and applying the triangle inequality, the probability gaps across the aforementioned sequence of games are cascaded and accumulated as follows:


$$\begin{array}{c} \frac{\text{1}}{\text{2}}\cdot {\text{Adv}}_{A}^{\text{AKA}}=\left|\text{Pr}\left[W_{\text{0}}\right]-\frac{\text{1}}{\text{2}}\right| \end{array}$$

$$\begin{array}{c} =\left|\text{Pr}\left[W_{\text{1}}\right]-\text{Pr}\left[W_{\text{4}}\right]\right| \end{array}$$

$$\begin{array}{c} \leq \left|\text{Pr}\left[W_{\text{1}}\right]-\text{Pr}\left[W_{\text{2}}\right]\right|+\left|\text{Pr}\left[W_{\text{2}}\right]-\text{Pr}\left[W_{\text{3}}\right]\right|+\left|\text{Pr}\left[W_{\text{3}}\right]-\text{Pr}\left[W_{\text{4}}\right]\right| \end{array}$$

$$\begin{array}{c} \leq \frac{q_{h}^{\text{2}}+q_{s}^{\text{2}}}{\text{2}|\text{Hash}|}+{\text{Adv}}_{A}^{\text{PUF}}+{\text{Adv}}_{A}^{\text{ECDLP}} \end{array}$$ (37)

Multiplying both sides of the inequality by 2 derives the final security probability bound relationship:


$$\begin{array}{c} {\text{Adv}}_{A}^{AKA}\leq \frac{q_{h}^{\text{2}}+q_{s}^{\text{2}}}{\left|Hash\right|}+\text{2}\cdot {\text{Adv}}_{A}^{PUF}+\text{2}\cdot {\text{Adv}}_{A}^{ECDLP} \end{array}$$ (38)

Under standard cryptographic assumptions, the ECDLP is widely recognized as unsolvable within polynomial time with a non-negligible probability, and the underlying physical secure domain possesses robust resistance against firmware extraction. Consequently, all advantage terms on the right side of the aforementioned security bound inequality represent negligible upper-bound functions within polynomial time, meaning that no PPT adversary can break the semantic security of the session key for the proposed scheme with a non-negligible advantage. In conclusion, the proposed scheme satisfies the semantic security of the session key and the existential unforgeability of anonymous authentication in the ROR model.

### 5.2 Proverif verification

ProVerif is an automated cryptographic protocol analysis tool widely applied to the detection of protocol logical flaws and security proofs under the Dolev-Yao adversary model. This section utilizes the ProVerif tool to conduct formal modeling and verification of the proposed scheme to evaluate its adaptive attack resistance in VANET environments.

To precisely monitor the state transitions and asymmetric identity binding between the vehicle and the RSU, four key security events are first formally defined in ProVerif to characterize the generation and verification of the NIZK, the initiation of vehicle authentication, and the negotiation of the session key. Subsequently, the underlying security of the scheme is comprehensively validated by introducing query statements for secrecy and strong mutual authentication with replay resistance. The security verification results of the proposed scheme are illustrated in Fig 7.

**Fig 7:**
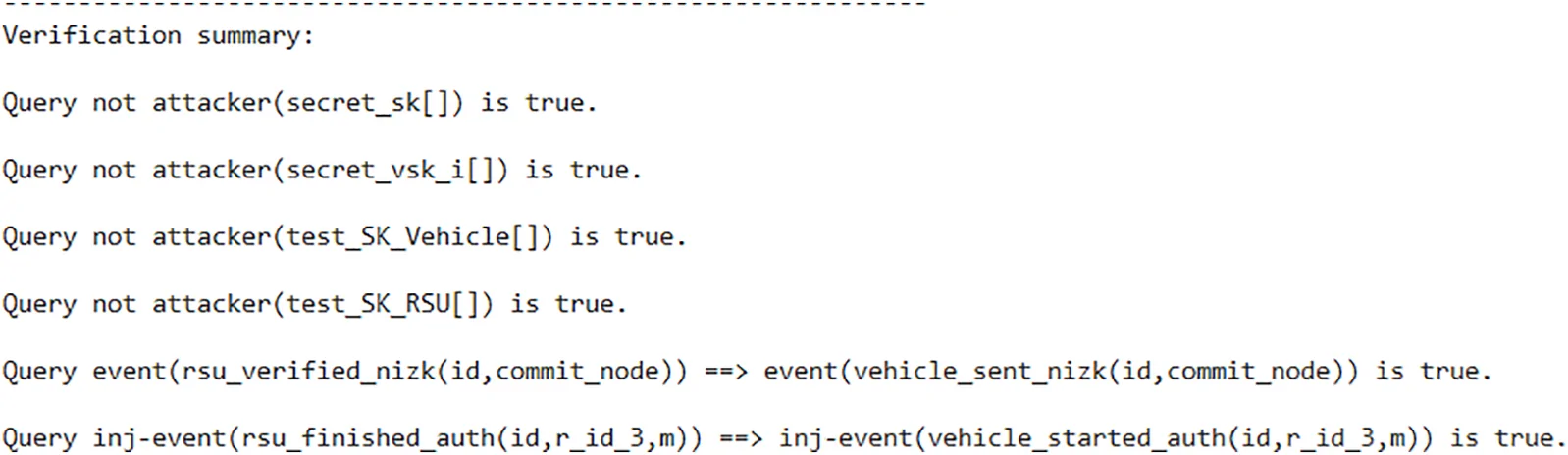
ProVerif security verification results.

First, regarding the verification of key secrecy, the verification results demonstrate that the secrecy queries for the system-wide master private key, the static and dynamic secret parameters of the vehicle, and the final shared session key generated by both communicating parties successfully pass the verification (all return true). This strongly proves that under the Dolev-Yao adversary model, even if an attacker completely controls the harsh wireless channel and eavesdrops on all public parameters or interactive messages, it remains impossible to reversely derive any entity’s private key within polynomial time. Simultaneously, this confirms that the session key computed by the vehicle and the RSU possesses complete semantic confidentiality, which effectively guarantees the secrecy of subsequent communications between both parties and prevents historical sessions from being maliciously compromised.

Second, in terms of entity authentication and security mechanism validation, the event correspondence queries for the zero-knowledge proof and the strong mutual authentication queries for the entities all smoothly pass the verification (all return true). Specifically, the satisfaction of the event correspondence queries confirms that the successful verification of the NIZK challenge equation at the RSU side depends entirely on the Pedersen commitment previously generated locally and transmitted to the channel by a legitimate vehicle. This implies that, in the absence of legitimate credentials and private keys, an attacker cannot forge an authentication request out of thin air that can pass the verification within polynomial time. Furthermore, the success of strong mutual authentication indicates a tight causal binding and cardinality agreement between the RSU’s acceptance decision and the vehicle’s session request. This defense mechanism eliminates the possibility of attackers constructing cross-session attack channels via concurrent multi-sessions, thereby proving that the proposed scheme is immune to any form of message replay and concurrent session attacks.

In conclusion, the verification results formally demonstrate that under the protection of the certificateless architecture and the zero-knowledge proof mechanism, the proposed scheme can effectively block man-in-the-middle attacks, impersonation attacks, and various replay attacks, perfectly achieving secure communication and conditional privacy-preserving in VANET environments.

### 5.3 Informal security analysis

Under the Dolev-Yao threat model, this section further demonstrates, through non-formal analysis, that the method proposed in the paper satisfies the core security requirements in the VANETs environment, including message authentication, identity privacy protection, anonymity, non-repudiation, unlinkability, and the ability to effectively resist various traditional attacks such as replay, forgery, collusion and consortium blockchain majority attacks. The specific analysis is as follows:

1. **Message Authentication and Integrity.** The proposed method ensures the authenticity and integrity of the message source using NIZK. In the authentication request $Req=({CID}_{new},E_{\text{1}},E_{\text{2}},R_{\text{1}},R_{\text{2}},C,T_{z})$, the challenge value $C=H_{\text{2}}(D∥{CID}_{new}∥E_{\text{1}}∥E_{\text{2}}∥T_{z})$ strictly binds all session parameters and the current timestamp. The vehicle constructs the response values $R_{\text{1}},R_{\text{2}}$ using its full private key ${vsk}_{i}$. Any tampering with ${CID}_{new}$, $E_{\text{1}}$, $E_{\text{2}}$, or the timestamp on the public channel by an adversary will cause a cascading effect on the challenge value C, thereby causing the verification equation at the receiving RSU to fail, fundamentally ensuring the authenticity of the message is not tampered with.
2. **Identity Privacy Protection.** The method achieves strong identity hiding. The vehicle does not directly transmit its real identity ${VID}_{i}$ or static composite identity ${CID}_{i}$. First, the real identity is concealed within the static commitment value $Commit={VID}_{i}\cdot G+r\cdot H$, which is based on the ECDLP. Second, the Commit is not sent in plaintext, but is blinded using the public key of the target RSU, generating the ciphertext ${(E}_{\text{1}},E_{\text{2}})$. This ensures that no third party, including other vehicles and eavesdroppers, can access the vehicle’s real identity, except for the target RSU that possesses the private key, thereby achieving perfect privacy protection.
3. **Anonymity and Unlinkability.** To prevent adversary multi-session tracking attacks, the method achieves strong unlinkability. Each time authentication is initiated, the vehicle selects a new temporary random number $r^{*}$ to generate a dynamic identifier ${CID}_{new}=Commit+r^{*}\cdot H$. Additionally, a new blinding factor ρ is introduced during the computation of the ciphertext ${(E}_{\text{1}},E_{\text{2}})$. Due to the involvement of random numbers, the authentication ciphertexts initiated by the same vehicle at different times and locations are probabilistically independent. The adversary cannot correlate the vehicle’s trajectory by comparing data packets from different sessions, effectively preventing identity tracking.
4. **Non-Repudiation.** The method provides non-repudiation. Since the response values $R_{\text{1}}$ and $R_{\text{2}}$ in the NIZK are bound to the vehicle’s full private key ${vsk}_{i}={vsk}_{i\text{1}}+t_{i}$, and $t_{i}$ is a secret component generated locally by the vehicle, the vehicle cannot deny sending a legitimate authentication request.
5. **Resistance to Common Attacks.** The proposed method effectively resists forgery, replay, and collusion attacks in VANETs. For forgery attacks, if an adversary attempts to forge a valid Req, they must solve the ECDLP problem in order to construct the response values $R_{\text{1}},R_{\text{2}}$ without knowing the private key ${vsk}_{i}$, which is computationally infeasible in polynomial time. For replay attacks, the challenge value C in the authentication request binds the current timestamp $T_{z}$. Upon receiving the request, the RSU first checks the timestamp. Any expired intercepted message is discarded directly, and even if an adversary attempts to modify the timestamp, it will break the hash verification. For collusion attacks, Since the full private key of the vehicle contains the locally generated secret component $t_{i}$, even if a malicious RSU colludes with the PKG, they cannot reconstruct the full private key of a legitimate vehicle, thereby ensuring the system’s security against collusion attacks.
6. **Resilience against Consortium Blockchain Collusion.** The proposed scheme guarantees that the system maintains robust security even under hostile scenarios where the consortium blockchain suffers from malicious collusion, majority attacks, or the hijacking of partial validator nodes. Specifically, since the consortium blockchain is utilized solely as a decentralized, read-only distributed ledger and does not directly participate in real-time online NIZK authentication or dynamic key agreement computations, this architectural decoupling effectively restricts the threat boundary of blockchain failures. Even when facing majority attacks or malicious collusion among validator nodes, where an adversary might attempt to tamper with historical block data or maliciously roll back the revocation status of vehicles by launching fork attacks, the real identity $VID_{i}$ of the vehicle remains concealed within the Pedersen commitment $Commit=VID_{i}\cdot G+r\cdot H$, which is blindly encrypted into the ciphertext pair $\left(E_{\text{1}},E_{\text{2}}\right)$ using the public key of the target RSU during transmission. Consequently, even if the majority of the consortium blockchain nodes are hijacked, malicious validators still cannot decrypt the authentication messages, nor can they trace the identities and driving trajectories of the vehicles.

## 6. Experimental evaluation

### 6.1 Experimental setup

Since the system initialization and vehicle registration phases are executed only once during the system lifecycle, whereas identity authentication and key agreement are high-frequency operations performed in every communication session, their performance directly determines the practicality and efficiency of the method. Therefore, this section focuses the performance analysis on the identity authentication and key agreement phase, and provides a detailed evaluation of the proposed method from the perspectives of computational overhead and communication overhead, with comparisons to other methods. Regarding the blockchain, in the proposed method, the generation and on-chain storage of vehicle static anonymous identities, as well as the uploading of system parameters to the blockchain, are all low-frequency tasks. The resulting transaction packaging delay or Gas overhead does not affect real-time communication. In the high-frequency end-to-end real-time authentication phase, the RSU only needs to query the blockchain to obtain the vehicle’s static commitment value. Blockchain queries do not involve node consensus or block packaging, and their latency is low, thus having little impact on the total end-to-end authentication delay.

To evaluate the computational overhead of the proposed method, simulation experiments were conducted. The experimental environment consisted of a computer equipped with an Intel Core i5-13400F @ 2.50 GHz processor and 16 GB of memory, running the Windows 11 operating system. The core cryptographic operations of the method were implemented using the Crypto library and the ecdsa library in Python, and SHA-256 was adopted as the hash function. This paper implements the security method based on standard ECC, using the additive group G of order q over the elliptic curve $y^{\text{2}}=x^{\text{3}}+ax+b\;$, with generator P. For the construction and testing of the blockchain network environment, this experiment employed Ganache to build a local Ethereum test network, so as to simulate the distributed ledger and node consensus environment in VANETs. The smart contracts in the system were written in Solidity (version 0.8.0), covering core logic such as RSU registration, vehicle credential writing, and CRL status query, and were compiled, tested, and locally deployed using the Truffle development framework. In the delay performance evaluation, the experiments were conducted based on Python’s web3.py library to interact efficiently with the underlying blockchain nodes through the HTTP RPC interface. The experiments separately performed 50 independent repeated tests for vehicle information query (read operation) and malicious vehicle revocation (write operation).

Based on standard elliptic curves, the paper benchmarks four types of fundamental cryptographic operations. For the elliptic curve scalar multiplication operation, the testing program randomly generates 1,000 scalars within the range [1,q−1] (large integers), and each scalar is multiplied with the fixed generator P. For the elliptic curve point addition operation, the testing program first generates two random scalars to calculate the corresponding two elliptic curve points, and then performs point addition on these two random points. For the SHA-256 hash operation, the testing program computes the hash value for 1,000 predefined formatted messages (e.g., “Test message 0001”). For the bilinear pairing operation, based on the bilinear map ${e:G}_{\text{1}}\times G_{\text{1}}\to G_{\text{2}}$, the testing program randomly selects two elements in the group $G_{\text{1}}$, performs the bilinear pairing map operation, and obtains the target element in the group $G_{\text{2}}$. Each of these operations is timed independently, and the final results are averaged over 1,000 runs. Partial experimental data are shown in Fig 8.

**Fig 8:**
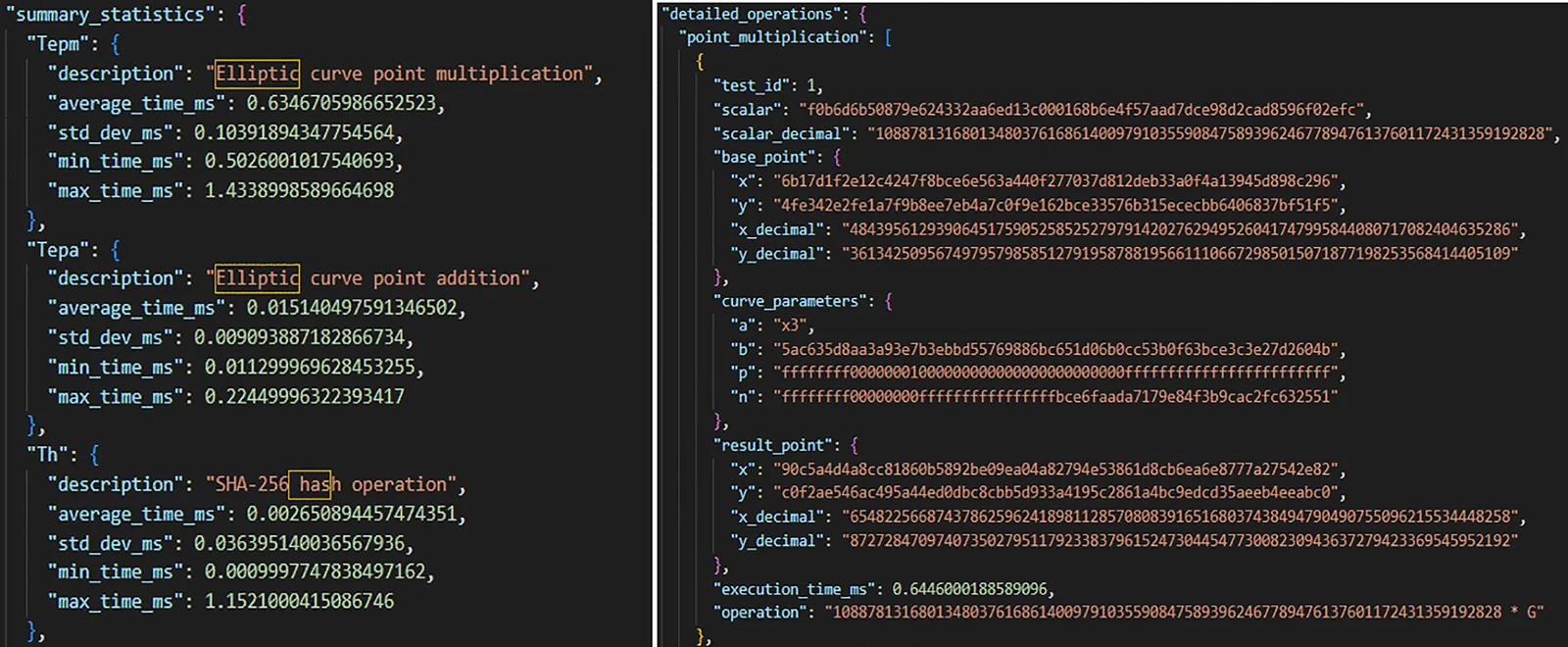
Experimental data samples.

To comprehensively evaluate performance, the paper selects five VANET security methods based on CL-PKC or blockchain technology. These include methods based on bilinear pairing mappings (as described in references [18] and [30]) and those based on standard ECC (as outlined in references [19–21] and [31]), allowing for a systematic verification of the relative advantages of the proposed method. The core of the proposed method’s design lies in developing a VANET privacy-preserving identity authentication and key agreement method based on CL-PKC and NIZK. This method ensures high-strength security attributes while enhancing computational and communication efficiency. Specifically, the proposed method uses Pedersen commitments to construct a dynamic anonymous identity generation mechanism. It stores the static identity on the blockchain and, during each authentication, generates a one-time dynamic pseudonym through re-randomization. This effectively addresses certificate management issues and ensures anonymity. In the authentication process, the method integrates private key validity verification and dynamic identity verification, enabling the vehicle to construct a single zero-knowledge proof to complete both verifications simultaneously. This approach not only eliminates key escrow risks but also avoids the high-cost computations of bilinear pairings, reducing the method’s interaction burden and computational latency. As a result, it is particularly well-suited for highly dynamic, resource-constrained VANET communication environments.

### 6.2 Experiments and results analysis

#### 6.2.1 Analysis of blockchain Gas cost and latency.

To evaluate the economic efficiency and computational overhead of the proposed method during blockchain deployment, the paper tested the Gas consumption of each core function in the smart contract. As shown in Fig 9, the costs of different operations exhibit significant differences. Among them, the Gas consumption of vehicle registration and RSU registration is relatively high, reaching 37919 Gas and 32797 Gas, respectively. This is because the registration phase requires the initialization of new storage slots in the Ethereum global state tree to permanently record key parameters such as the vehicle’s composite identity identifier (CID), public key components, and static commitment value. In contrast, the Gas costs of vehicle revocation and key agreement auditing are significantly lower, at only 24415 Gas and 24350 Gas, respectively. This is because the revocation and auditing processes only need to modify the state of existing mapping tables and do not require the writing of large-scale data. Overall, the proposed method uses cryptographic hashes and commitment values as on-chain credentials, thereby avoiding the direct uploading of raw plaintext vehicle data to the blockchain, reducing on-chain storage requirements, and enhancing system security. The Gas costs of all operations remain within a reasonable range, demonstrating the lightweight characteristics of the proposed method in terms of computation and storage resources, and enabling it to effectively satisfy the low-cost deployment requirements of VANETs.

**Fig 9:**
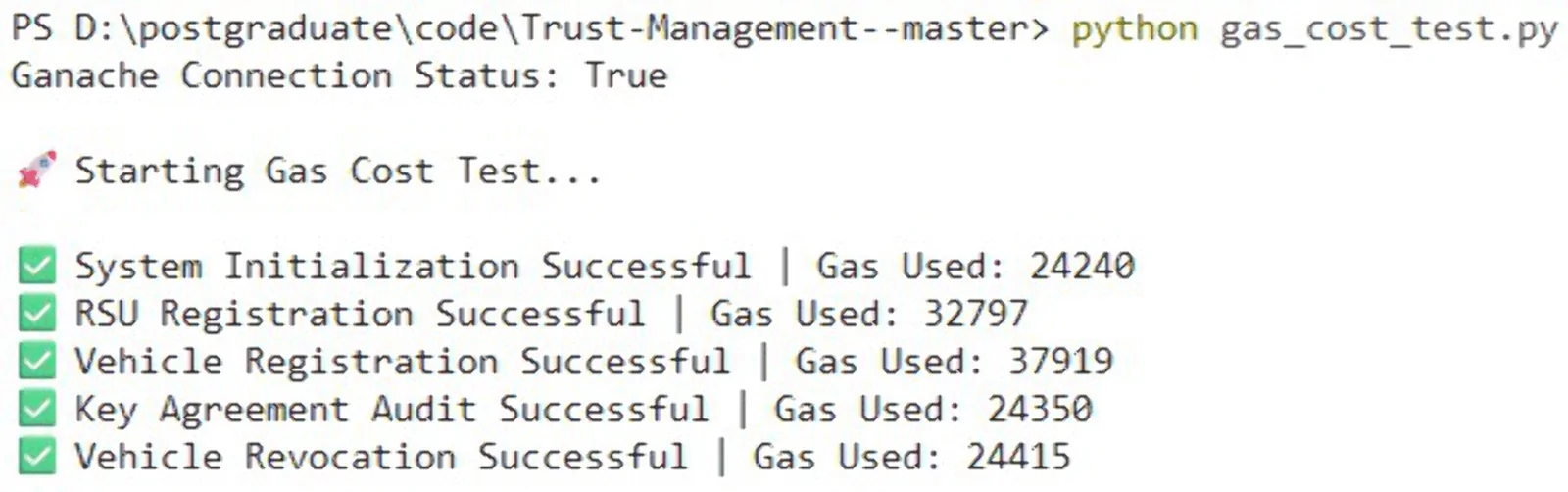
Gas costs at each stage.

In VANETs, the real-time performance of vehicle identity authentication is of critical importance. Therefore, the paper compares the latency distribution characteristics of read operations (state queries) and write operations (state changes). As shown in Fig 10, the proposed method exhibits good response efficiency in terms of latency performance. The average latency for querying vehicle registration information is relatively low, at approximately 14.61 ms, and the interquartile range of the data is very compact, indicating that the query operation is not only fast but also relatively stable in performance. This is because the query operation is a read-only call on the local node and does not involve complex cryptographic signature verification, transaction packaging, or network consensus. In contrast, the average latency of the write operation (malicious vehicle revocation) increases to approximately 22.23 ms, and the box is slightly wider. This phenomenon is consistent with the operating mechanism of blockchain systems. As a state-changing transaction, the write operation must go through core steps such as transaction packaging, state updating, and on-chain storage, making its processing procedure more complex than that of a read-only query. The very small number of outliers above the write operation in the figure, with the maximum reaching approximately 26.5 ms, reflects occasional instantaneous latency fluctuations in network request processing under a simulated high-concurrency environment. Nevertheless, the overall latency remains at a low level, fully demonstrating the real-time performance and feasibility of the proposed method for malicious vehicle tracking and rapid permission revocation in VANETs.

**Fig 10:**
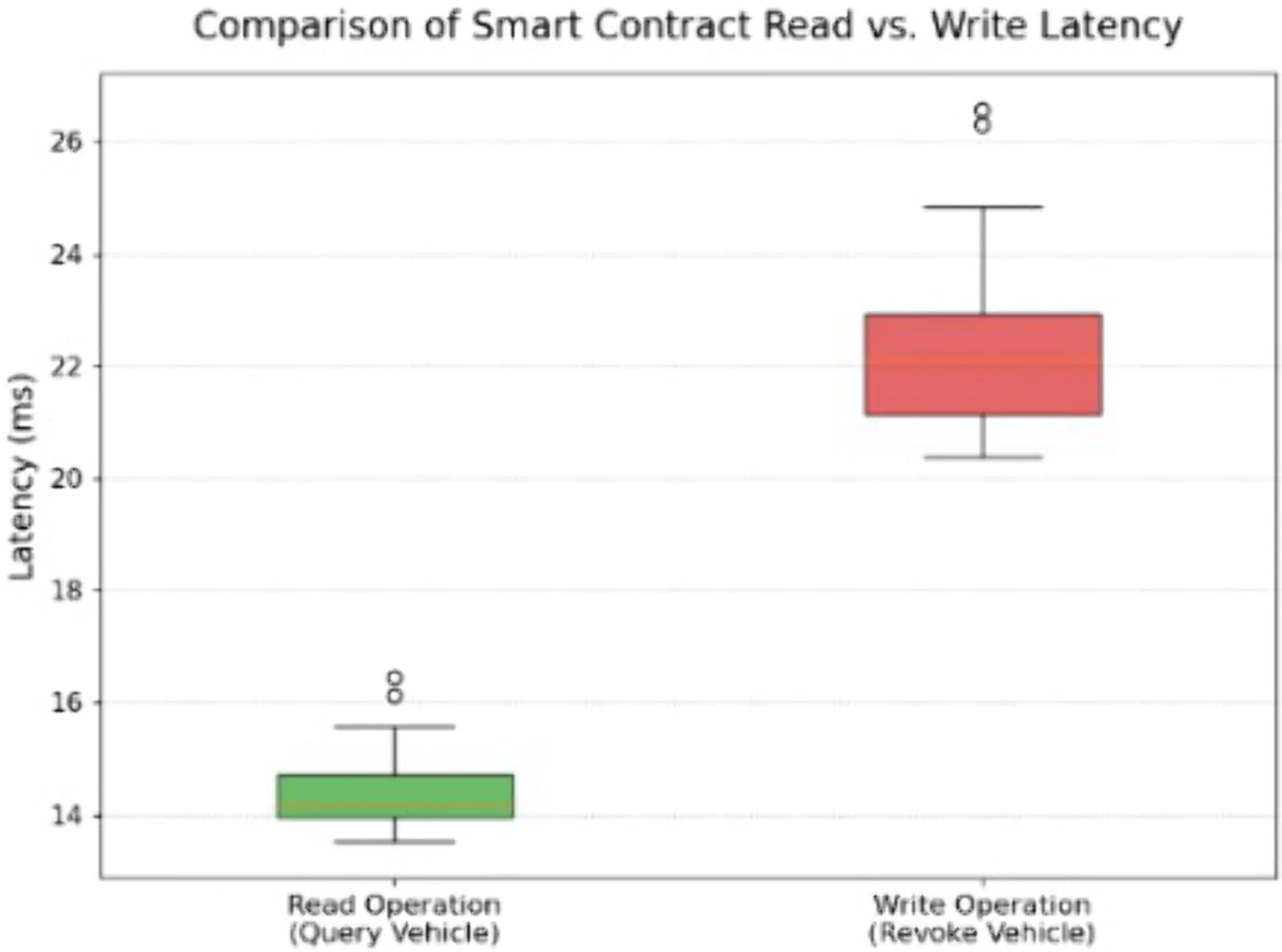
Comparative analysis of read and write latency.

#### 6.2.2 Computational overhead analysis.

The computational overhead of the proposed method’s authentication and key exchange phases is primarily distributed between the RSU and vehicle entities; thus, the focus is on their computational demands. To evaluate accuracy, 1000 encryption operations were executed, and the results were averaged to obtain the final outcome, which serves as the foundation for subsequent computational overhead assessments. The execution times for the cryptographic operations defined in the paper are provided in Table 2. Experimental findings indicate that the average time per operation for scalar multiplication on the elliptic curve group (Tepm) was 0.634 milliseconds, while point addition on the elliptic curve group (Tepa) averaged 0.015 milliseconds, the SHA-256 hash operation (Th) averaged 0.002 milliseconds, and the bilinear pairing operation (Tp) took 5.491 milliseconds. Since the computational overhead of hash functions is significantly lower than that of other basic operations, these operations were omitted in the paper to simplify the computational cost evaluation.

**Table 2:** Execution time of related operations.

Symbol	Description	Time(ms)
**Tepm**	Scalar Multiplication Operation on Elliptic Curve Group	0.634
**Tepa**	Point Addition Operation on Elliptic Curve Group	0.015
**Th**	SHA-256 hash operation	0.002
**Tp**	the bilinear pairing operation	5.491

To comprehensively evaluate the performance advantages of the proposed method, its anonymous authentication and key agreement approach is compared with the methods from [18–21,30,31]. The evaluation results are presented in Table 3, which reveals significant differences in computational overhead between the vehicle and RSU sides for the various methods.

**Table 3:** Computational overhead comparison.

Method	Vehicle Side	RSU Side	Total Time(ms)
**Method [18]**	Tepm	3Tp+Tepm	17.741
**Method [19]**	7Tepm	7Tepm	8.876
**Method [20]**	3Tepm+3Tepa	9Tepm+2Tepa	7.683
**Method [21]**	9Tepm+5Tepa	11Tepm+5Tepa	12.830
**Method [30]**	Tp + 8Tepm+3Tepa	Tp + 8Tepm+3Tepa	21.216
**Method [31]**	9Tepm+5Tepa	9Tepm+5Tepa	11.562
**Proposed Method**	6Tepm+3Tepa	7Tepm+6Tepa	8.377

In the proposed method, the vehicle side (OBU) requires 6 elliptic curve scalar multiplications and 3 point additions, totaling 6Tepm + 3Tepa. On the RSU side, 7 elliptic curve scalar multiplications and 6 point additions are required, totaling 7Tepm + 6Tepa. In the method presented in reference [18], although the vehicle side performs a lighter computation, requiring only 1 scalar multiplication, the RSU side needs to perform up to 3 bilinear pairing operations and 1 scalar multiplication, totaling 3Tp + Tepm. The high cost of bilinear pairings results in a total overhead of 17.741 ms, significantly higher than that of the proposed method. In reference [19], both sides perform 7 elliptic curve scalar multiplications, totaling 7Tepm for each side. The total execution time (8.876 ms) is slightly higher than the proposed method. In reference [20], the vehicle side requires 3 elliptic curve scalar multiplications and 3 point additions, totaling 3Tepm + 3Tepa, while the RSU side requires 9 elliptic curve scalar multiplications and 2 point additions, totaling 9Tepm + 2Tepa. The total computational overhead (7.683 ms) is slightly lower than the proposed method. In reference [21], the vehicle side performs 9 elliptic curve scalar multiplications and 5 point additions, totaling 9Tepm + 5Tepa, while the RSU side performs 11 elliptic curve scalar multiplications and 5 point additions, totaling 11Tepm + 5Tepa. The total computational overhead (12.830 ms) is notably higher than the proposed method. In the method presented in reference [30], both the vehicle side and RSU side each perform 1 bilinear pairing operation, 8 scalar multiplications, and 3 point additions, totaling Tp + 8Tepm + 3Tepa for each side. The bilinear pairing and numerous scalar multiplications increase the total computational overhead to 21.216 ms, making the computational costs on both the vehicle and RSU sides higher than the proposed method. Finally, In the method presented in reference [31], both the vehicle side and the RSU side require 9 elliptic curve scalar multiplications and 5 point additions, totaling 9Tepm + 5Tepa for each entity. Its total execution time reaches 11.562 ms, which is also higher than that of the proposed method due to the heavier algebraic burdens on both communicating sides.

Compared with the other six methods, the proposed method exhibits distinct advantages in computational efficiency. The methods in Refs. [18,30] adopt bilinear pairing cryptosystems, in which the core bilinear pairing operation incurs high computational overhead. As a result, their total computational costs, 21.216 ms and 17.741 ms, respectively, are significantly higher than those of ECC-based methods, making them difficult to satisfy the stringent real-time requirements of VANETs. The methods in Refs. [19,21,31] are also based on ECC; however, the communicating entities are required to perform more scalar multiplication operations, which increases the computational burden and leads to higher total execution times of 8.876 ms, 12.830 ms and 11.562ms, respectively. Although the method in Ref. [20] has a slightly lower total computational overhead (7.683 ms) than the proposed method, it relies only on a simple design based on basic elliptic curve scalar multiplication and does not incorporate a privacy-preserving mechanism at the level of NIZK. Meanwhile, although Ref. [31] avoids bilinear pairings, it lacks fine-grained security defense regarding cross-domain credential interactions and dynamic conditional privacy revocation. The comparison results of the total computational overhead of all methods are shown in Fig 11.

**Fig 11:**
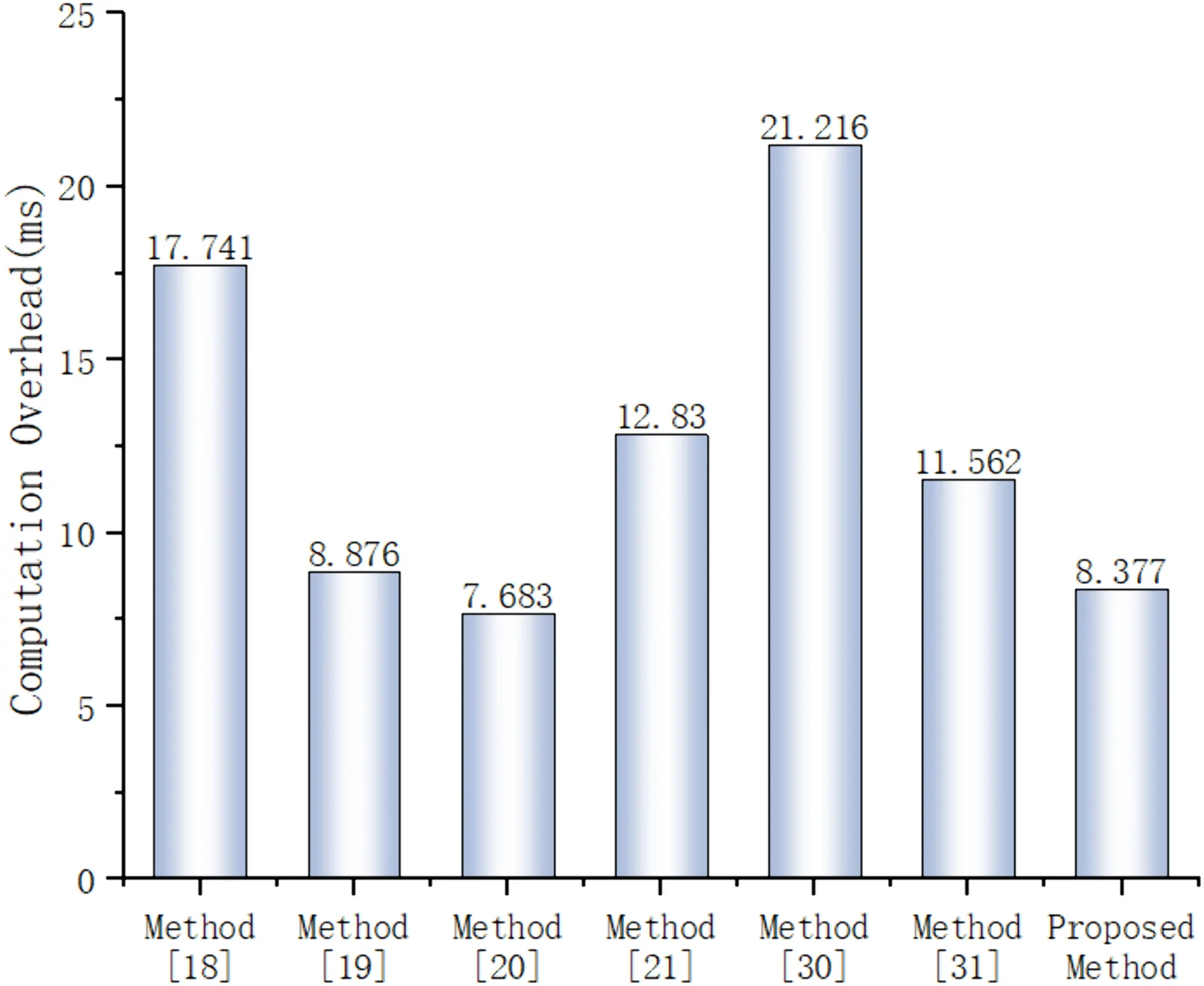
Total computational overhead.

As demonstrated in the experiments and shown in Figs 12 and 13, the experimental results show that the computational overhead of the proposed method on the vehicle side (OBU), approximately 3.849 ms, remains at a relatively low level and outperforms the methods in Refs. [19,21,30,31]. This superiority is primarily attributed to the fact that these baseline schemes accumulate excessive scalar multiplication operations on the onboard units. In the proposed method, the major computational tasks are assigned to roadside units with stronger computing capabilities, while the resource-constrained onboard units are responsible only for lightweight computations. This design enables the proposed method to maintain security while achieving a balance between security and computational overhead in terms of computational efficiency. In addition, another major reason why the proposed method maintains relatively high efficiency is that the adopted NIZK is an efficient variant based on the Schnorr protocol. It cleverly integrates the proof of ownership of the registered private key and the proof of the anonymous commitment into a single signature variant. This effectively avoids additional computational overhead during protocol execution and makes the method more suitable for VANETs environments.

**Fig 12:**
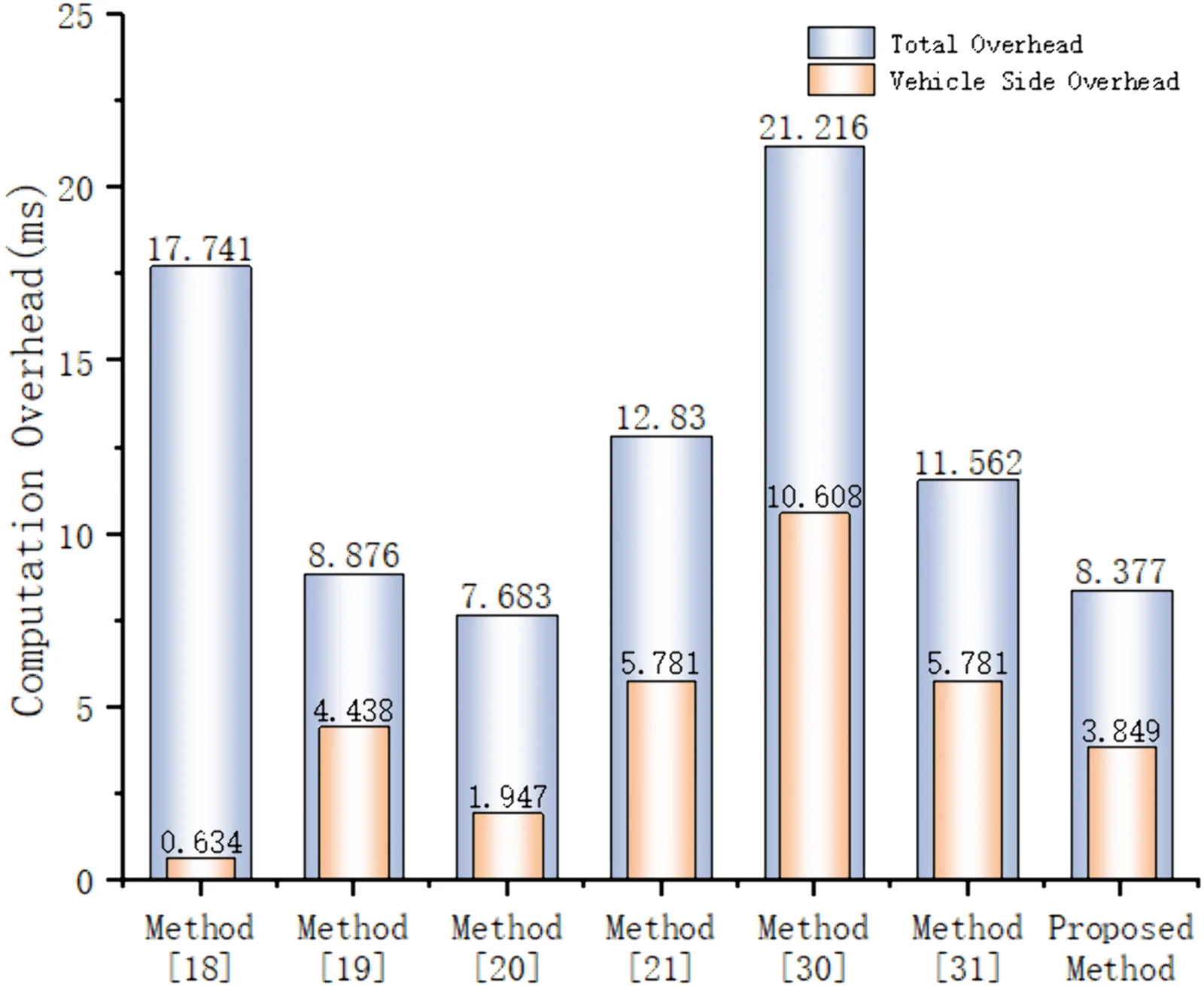
Comparison of vehicle-side and total computational overhead.

**Fig 13:**
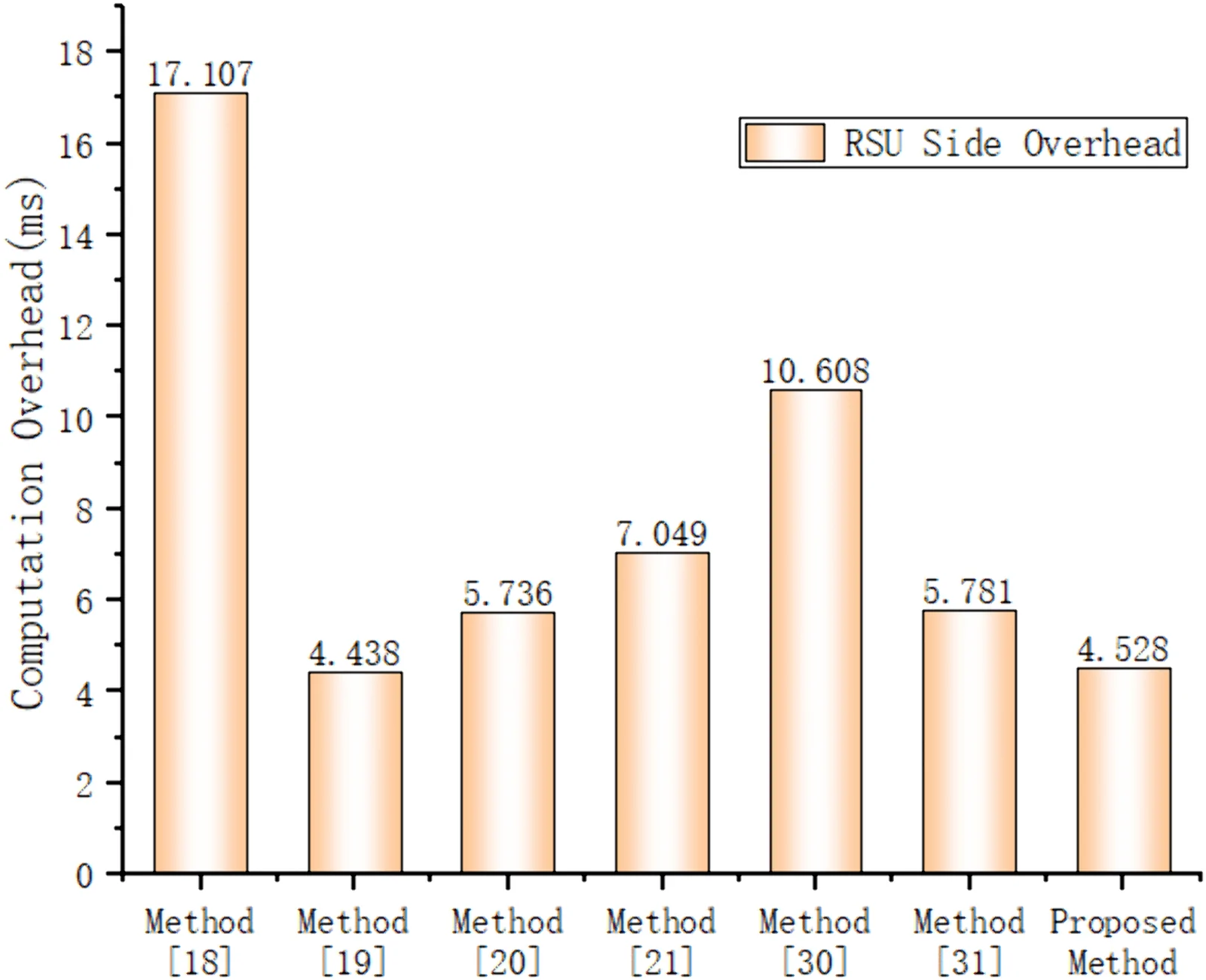
Computational overhead on RSU side.

Therefore, the proposed method not only achieves anonymous and verifiable identity authentication in terms of security, but also realizes a balance between security and computational overhead in terms of computational efficiency. Although its computation time is not the lowest, this moderate computational cost provides comprehensive security protection, including forward security, resistance to key leakage, and strong anonymity, making it particularly suitable for VANETs application scenarios with high security requirements.

#### 6.2.3 Communication overhead analysis.

The communication overhead for the authentication and key agreement phases of the proposed method is also primarily incurred between the RSU and the vehicle. Therefore, the analysis is focused on the communication overhead of these two entities. The bit lengths of the key data structures used for transmission are summarized in Table 4. The communication overhead during the authentication and key agreement phases for the four methods is evaluated based on the bit lengths provided in Table 4. This evaluation primarily considers the transmission of data structures including hash values (Lh), elliptic curve points (Lecc), identity identifiers (Lid), random numbers (Lran), and timestamps (Lts).

**Table 4:** Metadata lengths.

Symbol	Description	Length(bits)
**Lh**	Hash Value Length	256
**Lecc**	ECC Point Length	256
**Lid**	ID Length	256
**Lran**	Random Number Length.	256
**Lts**	Timestamp Length	64

To comprehensively evaluate the communication efficiency, the proposed authentication and key agreement method is compared with the methods from [19–21,30,31] in terms of communication overhead. The method in Ref. [18] involves only a single message transmission, achieving one-way identity authentication without session key agreement; therefore, it is excluded from the communication overhead comparison. The results are presented in Table 5. Significant differences are observed in the data volume transmitted during the communication process among the different methods.

**Table 5:** Communication overhead comparison.

Method	Vehicle Side	RSU Side	Total Length(bits)
**Method [19]**	3Lid + 2Lecc+Lh + 2Lts	2Lid+Lecc+Lh + Lts	2752
**Method [20]**	Lecc+2Lran+Lts	4Lecc+4Lran+2Lts	3008
**Method [21]**	Lecc+2Lran+Lts	5Lecc+4Lran+2Lts	3264
**Method [30]**	4Lecc+2Lran+Lts	4Lecc+2Lran+Lts	3200
**Method [31]**	2Lecc+Lran+Lid + Lts	2Lecc+Lran+2Lid + Lts	2432
**Proposed Method**	3Lecc+2Lran+Lh + Lts	Lecc+Lts	1920

In the method presented in Ref. [19], the messages exchanged between the vehicle and the roadside unit include elements such as identity identifiers, elliptic curve points, hash values, and timestamps, resulting in a total communication overhead of 2752 bits. In the methods presented in Refs. [20,21], the communication messages mainly consist of elliptic curve points, random numbers, and timestamps, with total communication overheads of 3264 bits and 3008 bits, respectively. In the method presented in Ref. [30], the communication messages likewise include elliptic curve points, random numbers, and timestamps, resulting in a total communication overhead of 3200 bits. Particularly, to guarantee a fair evaluation, for the cross-domain protocol in Ref. [31], only the message volume transmitted during its local vehicle-to-infrastructure (V2I) authentication session is calculated. In the local authentication phase of this method, the messages dispatched by the vehicle side and the RSU side contain elliptic curve points, random numbers, identity identifiers, and timestamps, yielding communication overheads of 1088 bits and 1344 bits, respectively, which accumulate to a total of 2432 bits.

In the identity authentication and key agreement process of the proposed method, only two messages are required. The first message, sent from the vehicle to the RSU, contains three elliptic curve points, two random numbers, one hash value, and one timestamp. The second message, sent from the RSU to the vehicle, contains only one elliptic curve point and one timestamp. The total communication overhead is 1920 bits, which is significantly lower than those of the other five methods.

The experimental results show that the proposed method exhibits clear advantages in communication efficiency, with an end-to-end total message size of only 1920 bits. Among the other compared methods, Ref. [21] has the highest total communication overhead, reaching 3264 bits. Refs. [20,30] follow, with 3200 bits and 3008 bits, respectively, while Ref. [19] has a total communication overhead of 2752 bits. Meanwhile, the communication overhead of Ref. [31] in local authentication is 2432 bits. By contrast, the proposed method achieves a total communication overhead of 1920 bits, representing reductions of approximately 41.2%, 40.0%, 36.2%, 30.2%, and 21.1% respectively, compared with these five methods. The comparison results of the total communication overhead among the six methods are shown in Fig 14.

**Fig 14:**
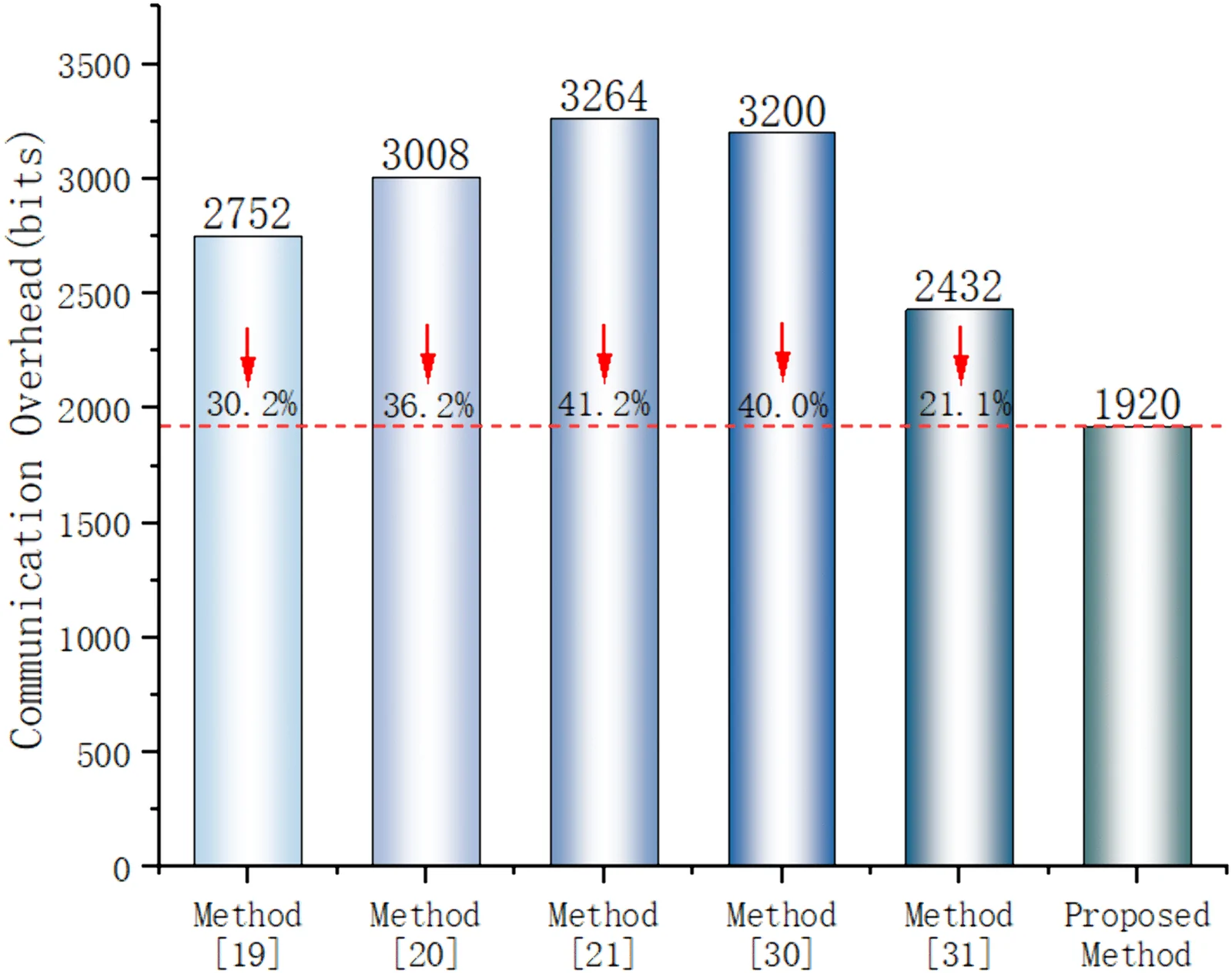
Total communication overhead.

Furthermore, in terms of the distribution of communication overhead between the vehicle and the RSU, the proposed method is more suitable for high-speed VANET scenarios. As shown in Figs 15 and 16, the proposed method places the majority of the communication burden (3Lecc + 2Lran + Lh + Lts) on the vehicle-initiated identity authentication request, while the RSU’s response is relatively lightweight (only Lecc + Lts). In high-speed VANET environments, the effective communication window between the vehicle and the RSU is often quite short. The proposed method reduces the data volume at the RSU side, allowing the entire authentication and key agreement process to be completed quickly within a short period, significantly reducing the risk of authentication failure due to channel delays or packet loss. In contrast, methods like those in Ref [21]. require the RSU to send a substantial amount of data (up to 5Lecc + 4Lran + 2Lts), which can have a considerable impact on communication performance in poor channel conditions or high-frequency handover scenarios.

**Fig 15:**
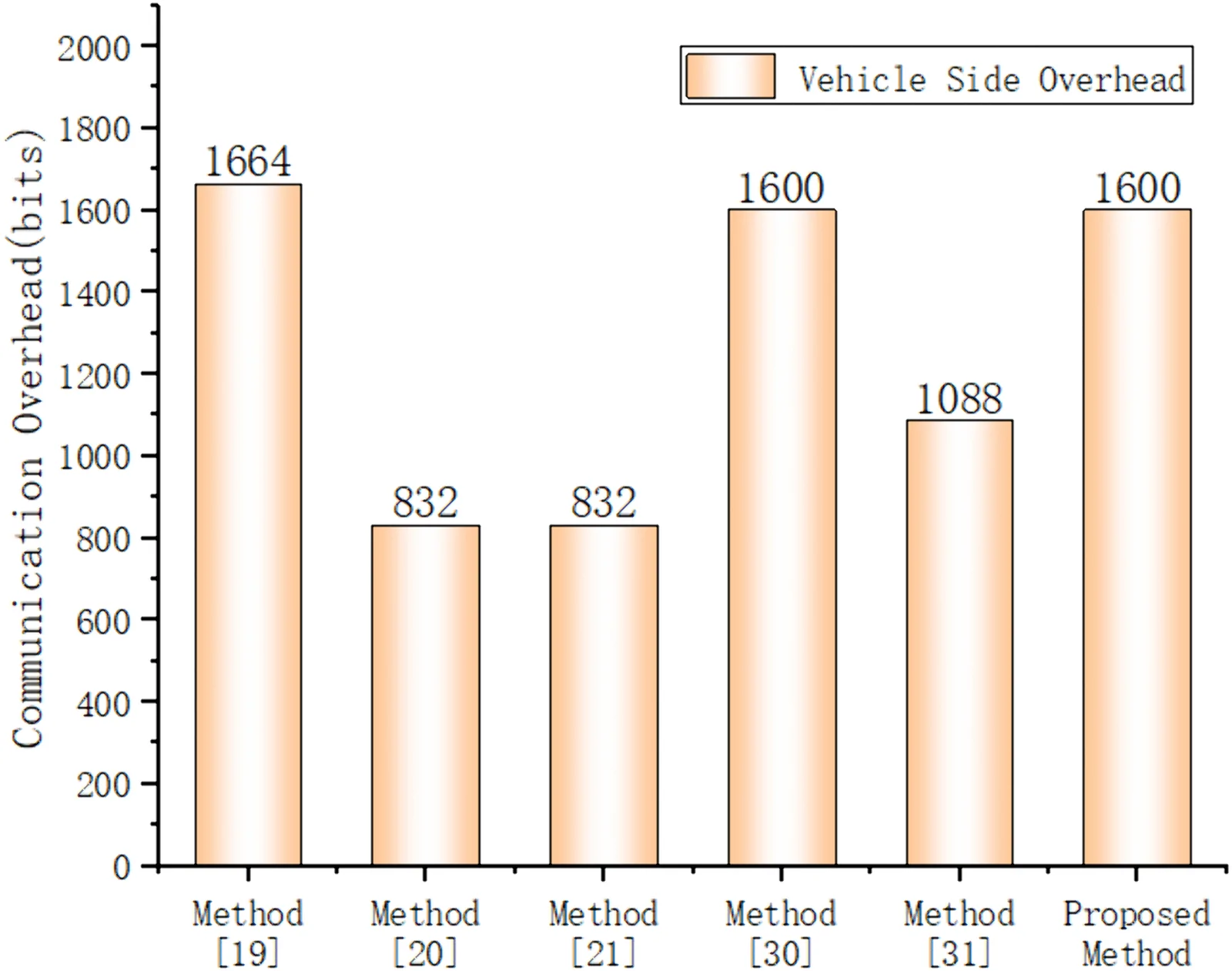
Vehicle-side communication overhead.

**Fig 16:**
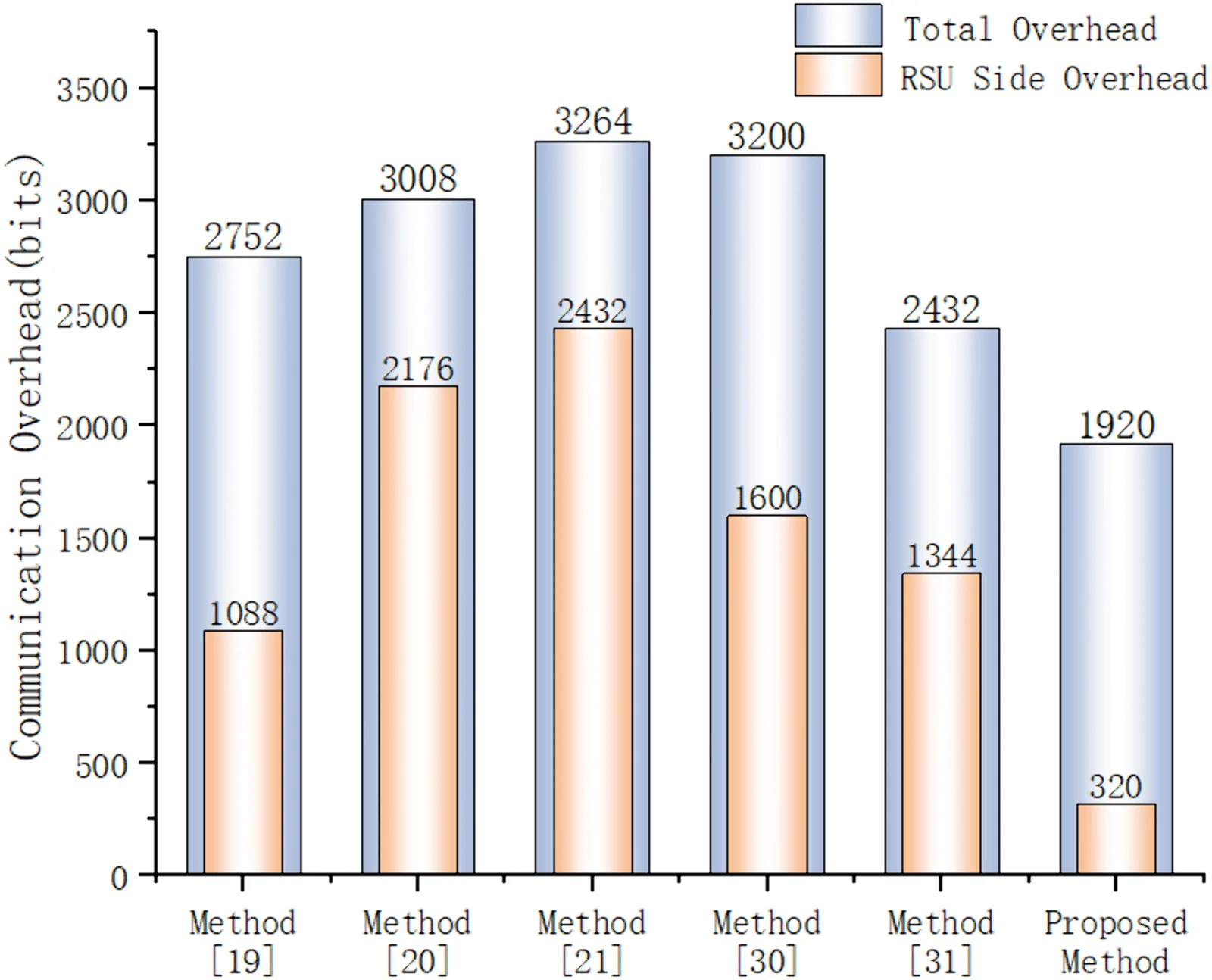
Comparison of RSU-side and total communication overhead.

The proposed method achieves relatively low communication overhead, with its core advantage lying in the efficient integration of CL-PKC and NIZK. The method employs a dynamic anonymous identity generation mechanism based on Pedersen commitments. During communication, the vehicle only needs to transmit a composite identity identifier generated through re-randomization. In addition, during authentication, the validity of the private key is combined with dynamic identity verification, allowing the vehicle to send a single request message to the RSU. A compact NIZK proof, based on the Schnorr protocol, enables the completion of dual verification simultaneously. This approach not only avoids the transmission of large signatures or ciphertext blocks but also reduces the traditional complex authentication dialogue to a single communication round. In summary, the proposed method strikes a balance between security and communication performance in terms of communication overhead.

### 6.3 Analysis of experimental results

This section provides a comprehensive comparison and analysis of various methods based on their security, computational, and communication overhead evaluations.

Overall, existing methods often struggle to achieve a good balance between computational efficiency, communication overhead, and privacy protection strength. For methods based on bilinear mappings (e.g., Refs. [18,30]), although they offer strong security guarantees, the bilinear pairing operations introduce significant performance overhead. The total computational overhead of the methods in Refs. [18,30] are 17.741 ms and 21.216 ms, respectively, with the communication overhead in Ref. [30] reaching 3200 bits. These substantial computational and communication costs make it difficult for these methods to adapt to high-dynamic, low-latency VANET environments. For methods based on standard elliptic curves (ECC) (e.g., Refs. [19,21]), while avoiding bilinear pairing operations, they still involve multiple point multiplication operations and additional parameter transmissions during authentication. The computational and communication overheads for Ref. [19] are 8.876 ms and 2752 bits, respectively, while the overheads for Ref. [21] are significantly higher, with computational time reaching 12.830 ms and communication overhead reaching 3264 bits. In contrast, the proposed method outperforms both in terms of computational and communication efficiency. For Ref. [20], the total computational time is 7.683 ms, slightly lower than the proposed method’s 8.377 ms, but the communication overhead is 3008 bits. Additionally, this method does not introduce a privacy protection mechanism at the level of NIZK, making it inadequate to meet the security requirements for strong anonymity and anti-forgery in VANETs. As for Ref. [31], although it targets cross-domain scenarios and avoids bilinear pairings, its local V2I communication overhead still reaches 2432 bits due to the transmission of multiple IDs and ECC points. Moreover, its total computational overhead is as high as 11.562 ms. More critically, it fails to integrate advanced privacy paradigms like NIZK, making it vulnerable to trackability under sophisticated adversary models.

Compared to other methods, the proposed approach achieves a better balance between security, computational overhead, and communication overhead. This is primarily due to the dynamic composite identity generation and authentication mechanism designed in this work. On the one hand, the proposed method integrates private key validity verification with dynamic identity authentication, simplifying the identity authentication process into a single round of interaction using an NIZK based on the Schnorr protocol. This effectively reduces the total communication overhead to 1920 bits. On the other hand, while ensuring security, the method reasonably allocates the main computational tasks to the more powerful roadside units (RSUs), thereby keeping the computational time at the vehicle onboard unit (OBU) side to 3.849 ms, which effectively alleviates the computational burden on resource-constrained devices.

In terms of end-to-end authentication latency, the cost of one complete identity authentication consists of communication transmission delay, the computational overhead of both parties, and the blockchain query delay at the RSU. The total authentication computation time of the proposed method is only 8.377 ms. Meanwhile, under modern VANET communication protocols, the channel transmission delay of 1920-bit communication data is less than 1 ms. In addition, the RSU query operation on the blockchain does not need to wait for block consensus and takes only 14.61 ms. In summary, the overall end-to-end authentication delay of the proposed method is low. While providing security properties such as forward security, identity privacy preservation, anonymity, and unlinkability, it can better satisfy the requirements of VANETs for high-frequency and highly real-time authentication.

## 7. Conclusion

To address the security issues in VANETs, such as the vulnerability of identity privacy to leakage, the complexity of certificate management, the high risk of key escrow, and the challenge of balancing authentication efficiency with security, the paper proposes a privacy-preserving identity authentication and key agreement method based on CL-PKC and NIZK. The method designs a dynamic anonymous identity generation mechanism by creating a composite identity identifier and combining it with Pedersen commitments. Static commitments containing identity information are stored on the blockchain, and a one-time dynamic identity is generated through re-randomization during each authentication, fundamentally addressing the certificate management complexity introduced by traditional pseudonym mechanisms and ensuring strong anonymity. Additionally, by integrating private key validity verification with dynamic identity verification, the vehicle only needs to use a single NIZK based on the Schnorr protocol to perform dual verification, effectively eliminating the risk of key escrow. Security analysis and experimental evaluation show that the proposed method not only resists various common attacks but also satisfies high-security requirements such as anonymity, unlinkability, and forward security. Compared to traditional methods based on PKI or IBC, the proposed method reduces both the certificate management burden and the system’s computational and communication overhead, making it suitable for highly dynamic and resource-constrained vehicular networks. However, given the extreme complexity of real VANET environments, massive high-concurrency authentication requests, limited computing capabilities of edge hardware such as onboard units (OBUs), and dynamic wireless communication channels may still pose challenges to the overall processing capability of the system. The current experiments in this paper mainly establish a cryptographic benchmark under a standardized computing environment to precisely verify the relative lightweight advantage of the proposed method. As a next step, further tests will be conducted in combination with real edge hardware architectures. Meanwhile, to evaluate the network scalability and protocol stability under dynamic topologies, large-scale simulation validation is planned within joint OMNeT++ and SUMO-based VANET environments, enabling the accurate measurement of realistic network performance indicators such as packet delivery ratio and end-to-end communication delay.
